# An electrogenetic interface to program mammalian gene expression by direct current

**DOI:** 10.1038/s42255-023-00850-7

**Published:** 2023-07-31

**Authors:** Jinbo Huang, Shuai Xue, Peter Buchmann, Ana Palma Teixeira, Martin Fussenegger

**Affiliations:** 1 Department of Biosystems Science and Engineering, ETH Zurich, Basel, Switzerland; 2 Faculty of Science, University of Basel, Basel, Switzerland

**Keywords:** Synthetic biology, Type 1 diabetes, Metabolism

## Abstract

Wearable electronic devices are playing a rapidly expanding role in the acquisition of individuals’ health data for personalized medical interventions; however, wearables cannot yet directly program gene-based therapies because of the lack of a direct electrogenetic interface. Here we provide the missing link by developing an electrogenetic interface that we call direct current (DC)-actuated regulation technology (DART), which enables electrode-mediated, time- and voltage-dependent transgene expression in human cells using DC from batteries. DART utilizes a DC supply to generate non-toxic levels of reactive oxygen species that act via a biosensor to reversibly fine-tune synthetic promoters. In a proof-of-concept study in a type 1 diabetic male mouse model, a once-daily transdermal stimulation of subcutaneously implanted microencapsulated engineered human cells by energized acupuncture needles (4.5 V DC for 10 s) stimulated insulin release and restored normoglycemia. We believe this technology will enable wearable electronic devices to directly program metabolic interventions.

## Abstract (web summary)

Huang et al. develop an interface to allow electrode-mediated stimulation of gene expression in human cells, utilizing direct current-generated reactive oxygen species to stimulate transgene expression downstream of the KEAP1–NRF2 biosensor. In a type 1 diabetic mouse model, this interface is demonstrated to ameliorate hyperglycemia by stimulating insulin expression.

## Main

Interconnected smart electronic devices are increasingly dominating our daily lives and shaping our health awareness^1^; however, electronic and biological systems function in radically different ways and are largely incompatible due to the lack of a functional communication interface. While biological systems are analog, programmed by genetics, updated slowly by evolution and controlled by ions flowing through insulated membranes, electronic systems are digital, programmed by readily updatable software and controlled by electrons flowing through insulated wires. Electrogenetic interfaces that would enable electronic devices to control gene expression remain the missing link in the path to full compatibility and interoperability of the electronic and genetic worlds^2^.

Synthetic biology has taken up this challenge by assembling simple analog gene switches into complex gene circuits that can program cellular behavior with the logic-processing functionality of electronic circuits such as oscillators^3^, timers^4^, memories^5^, band-pass filters^6^ and relay switches^7^ as well as analog-to-digital converters^8^, half-adders^9^ and even full-adders^10^. The utility of many of these gene circuits has been demonstrated in the experimental control of diverse medical conditions, including cancer^3^, bacterial infections^11^, chronic pain^12^ and diabetes^13^. Gene circuits typically incorporate trigger-inducible gene switches that are controlled by small-molecular compounds such as antibiotics^14^, vitamins^15^, food additives^16^, cosmetics^17^ or volatile fragrances^8^. As differences in bioavailability, pleiotropic side effects and pharmacodynamics may jeopardize the overall regulatory performance of such triggers in a mammalian host, attention has increasingly turned to non-molecular traceless physical cues such as electromagnetic waves, including light^18,19^, magnetic fields^20^, radio waves^21^ and heat^22^; however, physically triggered gene switches may require high energy input^21^, may involve unphysiological chemical or inorganic cofactors with side effects^19^, poor bioavailability^23^ or short half-lives^24^, may suffer from illumination-based cytotoxicity^25^ and may be confounded by any fever-associated medical condition^22^.

Thus, there is a need for a device to permit direct battery-powered, cofactor-free, time- and voltage-dependent electrical fine-tuning of mammalian gene expression to set the stage for wearable-based electro-controlled gene expression with the potential to connect medical interventions to an internet of the body or the internet of things. Pioneering attempts to design electro-inducible gene expression in bacteria^26–30^ and mammalian cells^31–33^ proved promising in cell cultures, but were either incompatible with in vivo applications due to the cytotoxicity, limited bioavailability and poor clinical compatibility of electrosensitive redox compounds^26,31^ or required high-voltage alternating current controlled by complex bioelectronic implants with limited longevity^32^. Such devices are not suitable for use in battery-powered wearables to program therapeutic transgene expression in implanted cells^32^.

In humans, reactive oxygen species (ROS) are produced by electron-transfer reactions during respiratory processes in the mitochondria and peroxisomes, during mitochondrial cytochrome P450 activity in steroidogenic tissues and by NADPH oxidase in immune cells during immune responses^34^. The Kelch-like ECH-associated protein 1 (KEAP1) is an important tumor and metastasis suppressor that also acts as a native ROS biosensor^35^. Under quiescent conditions, KEAP1 sequesters and primes the nuclear factor erythroid 2 p45-related factor 2 (NRF2) for proteasomal destruction^35^. In the presence of elevated ROS, KEAP1 releases NRF2, which translocates to the nucleus to coordinate antioxidant and anti-inflammatory responses by binding to antioxidant-response elements (AREs)^35^.

Inspired by the fact that electrodes delivering DC at low voltage can rapidly generate free electrons and radical species that lead to mediator-free production of ROS at low, non-cytotoxic levels^36–38^, we set out to design the missing link for DC-powered electrogenetic target gene modulation in human cells, which we refer to as DC-actuated regulation technology (DART). DC-based generation of hydrogen peroxide was recently applied to establish an electrogenetic system using engineered bacterial cells growing at the surface of an electrode, which was able to activate transgene expression upon electrical stimulation^30^. Here we designed an electrogenetic interface consisting of genetic components that render human cells responsive to DC-triggered electrostimulation and enable exclusive, DC-adjustable transgene expression. We initially found that the ROS levels produced in human embryonic kidney cells (HEK293) by exposure to 4.5 V DC for 10 s were not sufficient to substantially activate KEAP1/NRF2; however, the cells could be hypersensitized to electroinduced ROS by ectopic expression of native KEAP1 and NRF2. Then, rewiring of NRF2 to synthetic ARE-containing promoters enabled direct and cofactor-free DC-powered fine-tuning of the expression of therapeutic transgenes such as the insulin gene. For a proof of concept, we implemented DART-based remote control of insulin expression in type1 diabetic mice. Stimulation of subcutaneously implanted engineered cells with World Health Organization (WHO)-approved and US Food and Drug Administration (FDA)-licensed acupuncture needle electrodes at 4.5 V DC for 10 s once per day triggered the production of sufficient insulin to attenuate postprandial glycemic excursions and restore normoglycemia.

## Results

### Design and characterization of DC-controlled gene expression

**Fig. 1:**
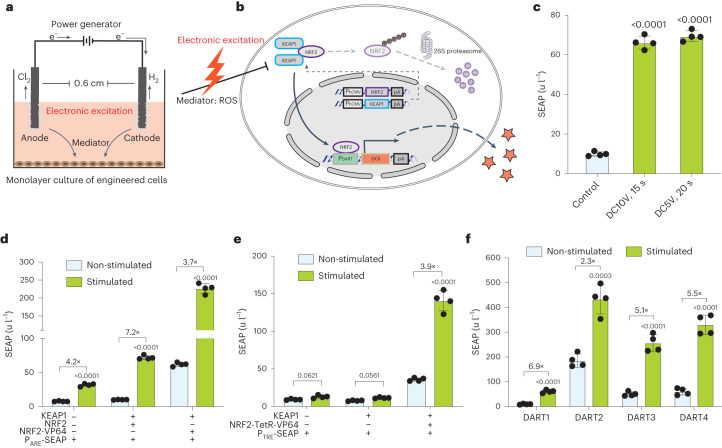
Design of the direct-current-activated transgene expression switch in mammalian cells. **a**, Schematic illustration of the stimulation setup for monolayer cultures. Each well of a 24-well plate has two platinum wires that function as anode and cathode, placed 0.6 cm apart submerged in the culture medium. When electric current is applied, bubbles form around the electrodes, with production of chlorine gas at the anode and hydrogen gas at the cathode. **b**, Schematic representation of the electrogenetic circuit based on the NRF2/KEAP1 antioxidative response. Upon electrical stimulation, the formation of ROS is sensed by constitutively expressed NRF2 and KEAP1 complexes localized in the cytoplasm, which triggers the translocation of NRF2 to the nucleus, where it activates expression of the gene of interest by binding to ARE sites in the upstream synthetic promoter. Under non-stimulating conditions, NRF2 is continuously targeted to the 26S proteasome for degradation. **c**, SEAP produced by transiently transfected HEK293 cells (KEAP1, pJH1004; NRF2, pJH1003; P_DART_-SEAP, pJH1005) upon stimulation by DC with 10 V for 15 s (DC10V) and 5 V for 20 s (DC5V). **d**, SEAP produced by cells transfected with only ARE reporter (P_DART_-SEAP, pJH1005) or together with KEAP1 (pJH1004) and NRF2 variants (wild-type NRF2, pJH1003; NRF2-VP64, pJH1175) and reporter (pJH1005). Cells were stimulated with DC5V for 20 s. **e**, SEAP produced by cells co-transfected with KEAP1 (pJH1004), NRF2 fused to tetracycline-dependent transactivator TetR-VP64 (NRF2-TetR-VP64, pJH1181) and the cognate reporter (P_TRE_-SEAP-pA, pMF111). The cells were stimulated with DC5V for 20 s. **f**, SEAP produced by cells co-transfected with reporter constructs containing one (DART1), two (DART2), three (DART3) and four (DART4) ARE repeats in the promoter region and stimulated with DC5V for 20 s. Data are mean ± s.d., *n* = 4. *P* values were calculated between stimulated and non-stimulated controls. Source data

To sensitize native human cells for electrostimulated ROS-mediated transgene expression control, we co-transfected human embryonic kidney cells (HEK293) with constitutive KEAP1 (pJH1004, P_hCMV_-KEAP1-pA) and NRF2 (pJH1003, P_hCMV_-NRF2-pA) expression vectors as well as the reporter construct pJH1005 (P_DART_-SEAP-pA; P_DART_, O_ARE_-P_hCMVmin_) encoding the human model glycoprotein SEAP (human placental secreted alkaline phosphatase) under the control of a synthetic NRF2-dependent promoter containing an ARE operator site (Supplementary Table 1). Protons and chlorine ions are generated in the cell culture medium at the electrodes^36,37^ (Fig. 1a) and lead to the production of ROS at levels^38^ that can trigger the release of NRF2 from KEAP1, resulting in NRF2-mediated expression of a gene of interest from the NRF2-specific synthetic P_DART_ promoter (Fig. 1b).

For electrostimulation, the engineered cells were cultivated in standard 24-well plates containing customized lids that serve to immerse 0.5-mm platinum electrodes in the culture medium at a separation distance of 6 mm (Fig. 1a, Supplementary Fig. 1a,b and Supplementary Video 1). We observed significantly increased SEAP expression upon electrostimulation with DC at 10 V for 15 s or 5 V for 20 s (Fig. 1c). Alternating current (AC) pulse programs delivering similar energy to these DC programs (for instance, 1-ms pulses of 5 V at 1 Hz during 2.78 h) resulted in a less than two-fold increase in SEAP expression and significantly decreased cell viability (Supplementary Figs. 1c–f and 2). Indeed, to reach comparable SEAP expression levels, AC stimulation needs to deliver more energy and run for longer time periods (6 h for 1-ms pulses (Supplementary Fig. 2c); or over 1 h for 10-ms pulses (Supplementary Fig. 2e)), again with a negative impact on cell viability (Supplementary Figs. 1c–f and 2). Therefore, hereafter we focused on DC as our sole energy source. Notably, DC stimulation programs below 5 V and 20 s had no impact on cell viability (Supplementary Figs. 2 and 3a), medium composition (Supplementary Fig. 3b–e and Supplementary Tables 2–5), growth kinetics or overall protein production capacity of stimulated cells (Supplementary Fig. 4), indicating that DC-mediated electrostimulation can indeed be specifically rewired to DART promoters to fine-tune transgene expression in human cells. Electrostimulated HEK293 cells exclusively transfected with pJH1005 showed substantially less SEAP induction, indicating that concomitant coexpression of KEAP1 and NRF2 increases the sensitivity of the human cells to electrostimulated ROS production (Fig. 1d). We also tested whether NRF2 fused to the strong transactivation domain VP64, which is based on four tandem repeats of the *Herpes simplex* virus early transcriptional activator VP16 (pJH1175, P_hCMV_-NRF2-VP64-pA), could further increase the electrostimulated response; however, this modification increased basal as well as electrostimulated SEAP expression, resulting in a substantially lower overall induction fold (3.7×) compared to native NRF2 (7.2×) (Fig. 1d). Likewise, we observed lower fold induction (3.9×) of NRF2 fused to the tetracycline-dependent transactivator TetR-VP64 (pJH1181, P_hCMV_-NRF2-TetR-VP64-pA) activating SEAP expression from a promoter containing tetracycline-response elements (TRE) (pMF111, P_TRE_-SEAP-pA; P_TRE_, O_TetR_-P_hCMVmin_) (Fig. 1e). Further analyses with P_DART_ variants containing different ARE tandem repeats revealed that a single ARE repeat provided low basal expression but also the lowest maximum expression level, while more tandem repeats achieved substantially higher maximum expression levels at the expense of higher basal expression (Fig. 1f). Therefore, the priority for either lowest leakiness or highest expression level will determine the choice of P_DART_ variants, as is also the case for other transcription-control modalities^39^.

**Extended Data Fig. 1:**
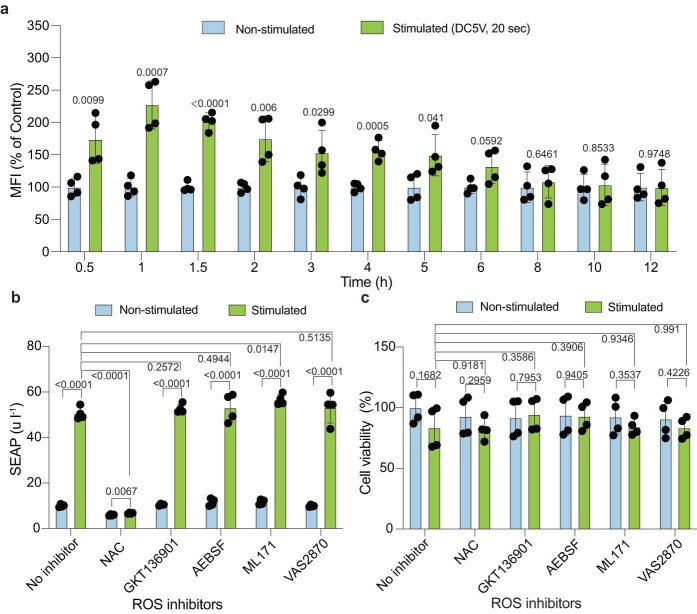
Characterization of the ROS-mediated synthetic gene switch. **a**, Time course analysis of intracellular ROS in DART-engineered HEK-293 cells after electrostimulation with DC 5 volts for 20 s. ROS levels were measured at indicated time points. MFI: mean fluorescence intensity. **b,c** Effect of ROS inhibitors on DART-engineered HEK-293 cells. SEAP production (**b**) and cell viability (**c**) 24 hours after electrostimulation with DC 5 volts for 20 s. The cells were incubated with the ROS scavenger *N*-acetyl-*L*-cysteine (NAC, 2 mM), or inhibitors of ROS-generating enzymes GKT136901 (0.2 μM), AEBSF (2 μM), ML171 (1 μM) and VAS2870 (2 μM) for 30 min before performing electrostimulation. Each bar represents the mean ± SD of the four indicated data points. *P* values were calculated between stimulated and non-stimulated or indicated group. Source data

**Extended Data Fig. 2:**
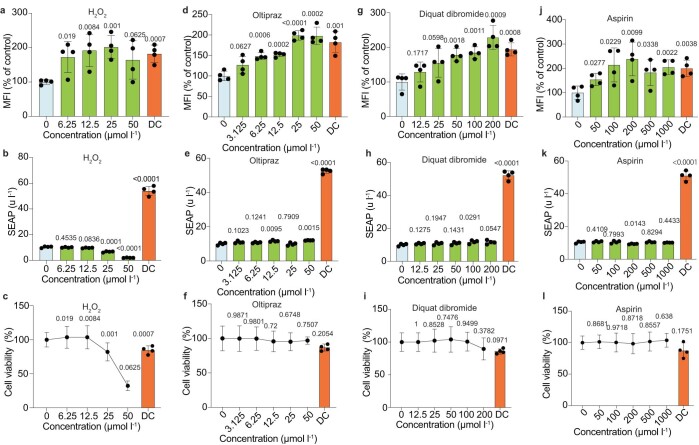
Effect of ROS-inducing chemicals on DART-engineered HEK-293 cells. **a-l**, ROS generation, SEAP production and cell viability were analyzed after treating the cells with H_2_O_2_ (**a-c**), oltipraz (**d-f**), diquat dibromide (**g-i**) or aspirin (**j-l**), at the indicated concentrations. Cell viability was measured by incubation with 50 μg/mL resazurin for 2 h at 37 °C. The samples marked as DC (orange bars) in all panels represent the positive control (stimulation at 5 volts DC for 20 s). Columns each represent the mean ± SD of the four indicated data points. Symbols in (**c**), (**f**), (**i**) and (**l**) represent the mean ± SD of 4 determinations. MFI, mean fluorescence intensity. *P* values were calculated between stimulated and non-stimulated group. Source data

**Extended Data Fig. 3:**
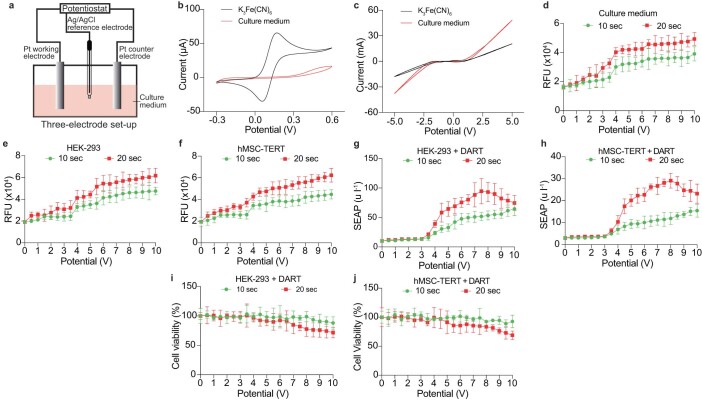
Analysis of ROS levels, SEAP production and cell viability when applying different half-cell potentials. **a**, Schematic model of the three-electrode set-up, with a potentiostat connected to an Ag/AgCl reference electrode and two platinum electrodes, of which one serves as a working electrode and the other as a counter-electrode. **b**,**c**, Cyclic voltammogram (CV) in standard potassium hexacyanoferrate (III) (K_3_Fe(CN)_6_) solution (10 mM, black) or in culture medium (DMEM plus 10% FBS, red) at the potential window of −0.3 to 0.6 V (**b**) and −5.0 to 5.0 V (**c**). All CVs were scanned at a rate of 10 mV s^−1^. **d**-**f**, ROS quantification in cell-free culture medium (**d**), DART-engineered HEK-293 cell cultures (**e**) and DART-engineered hMSC-TERT cell cultures (**f**). RFU, relative fluorescence units. **g**,**h**, SEAP produced by DART-engineered HEK-293 cells (**g**) and DART-engineered hMSC-TERT cells (**h**), 24 h after electrostimulation. **i**,**j**, Viability of DART-engineered HEK-293 cells (**i**) and DART-engineered hMSC-TERT cells (**j**), 24 h after electrostimulation. All stimulations were performed with the three-electrode system, during 10 or 20 sec at the indicated voltages. Data points represent mean ± SD; n = 4. Source data

**Extended Data Fig. 4:**
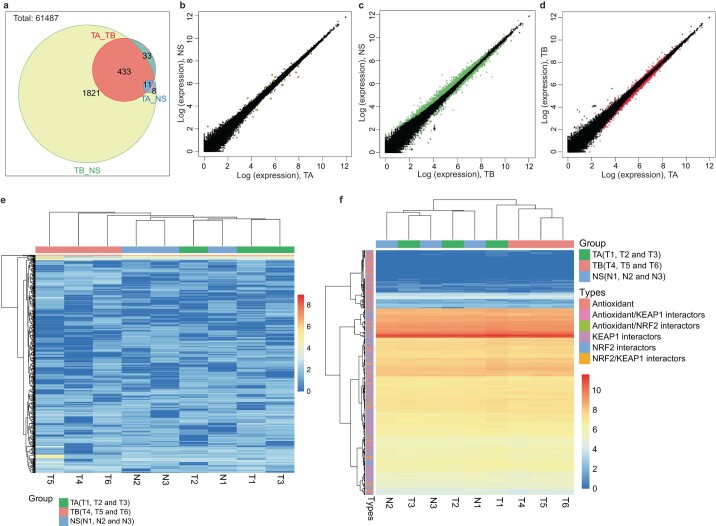
Gene expression analysis in non-stimulated and electrostimulated HEK-293 cells. **a**, Venn diagram of gene expression differences among non-stimulated cells (NS) and electrostimulated cells at 5 volts DC for 10 s (TA) or 25 s (TB). The total number of sequenced transcripts was 61487. **b**, Gene expression levels in TA versus NS. The differentially expressed transcripts are shown in orange. **c**, Gene expression levels in TB versus NS. The differentially expressed transcripts are shown in green. **d**, Gene expression levels in TA versus TB. **e**, Heatmap of the top 500 transcripts with largest standard deviation of expression counts among non-stimulated cells (NS) and electrostimulated cells at 5 volts DC for 10 s (TA) or 25 s (TB). Each group contains three replicates. The N1, N2 and N3 are the samples in NS group. The T1, T2 and T3 are the samples in TA group. The T4, T5 and T6 are the samples in TB group. The top 500 transcripts are listed orderly from highest to lowest in Supplementary List. The heapmap data were analyzed by PCAtools in R. **f**, Heatmap of genes of interest (450 genes). T1, T2 and T3 are highly clustered with N1, N2 and N3, respectively. The gene groups are indicated by colors. Heatmap annotations are the same as in (**e**). The details of clustering of each gene are listed orderly from top to bottom in Supplementary List. The differentially expressed transcripts are shown in red. Mean expression levels (n = 3) are shown as natural logarithms in (**b**), (**c**) and (**d**). In (**e**) and (**f**), the color in heatmap indicates the expression levels. The data represent log(e) of sequencing reads assigned to genes.

A fluorescence-based assay showed that intracellular ROS levels were increased over two-fold at 1 h after DC electrostimulation at 5 V for 20 s and returned to non-stimulated levels within 6 h, suggesting that electrostimulated SEAP expression was indeed mediated by ROS^36–38^ (Extended Data Fig. 1a). Notably, cells treated with the ROS scavenger *N*-acetyl-*L*-cysteine (NAC)^40^ failed to increase SEAP expression upon electrostimulation (Extended Data Fig. 1b,c). In contrast, cells treated with inhibitors of different ROS-generating enzymes, such as GKT136901, AEBSF, ML171 and VAS2870 (ref. ^41^), could still respond to electrostimulation by increasing SEAP expression (Extended Data Fig. 1b,c), supporting the view that the ROS generation results from the DC power stimulation rather than from endogenous sources. Furthermore, although hydrogen peroxide (H_2_O_2_), oltipraz, diquat dibromide^42^ and aspirin^43^ can induce ROS surges in cells, the DART system showed no SEAP response to these chemicals (Extended Data Fig. 2), suggesting that DART is not responsive to the free radicals generated by these chemicals (for instance, hydroxyl radicals (^•^OH) from H_2_O_2_ treatment^44^). Conversely, DC electrostimulation generates free electrons that can be received by different molecules to form a wider range of radicals and some of them can trigger the DART system by activating transgene expression from the synthetic P_DART_ promoter. We also analyzed the resulting current, the ROS levels, SEAP response and impact on cell viability when the applied DC voltages were measured in a three-electrode setup incorporating an Ag/AgCl reference electrode, thereby demonstrating the tunability of the DART system in vitro (Extended Data Fig. 3). Next-generation sequencing revealed that DC electrostimulation at 5 V for 10 s had negligible impact at the transcriptome level, but 5 V for 25 s had a slight effect, with a small set of genes mainly associated with antioxidant response being differentially expressed compared to non-stimulated cells (Extended Data Fig. 4 and Supplementary List). These results indicate that stimulation times <25 s do not interfere with DART control and all follow-up experiments were conducted accordingly.

### Characterization and validation of DART

**Fig. 2:**
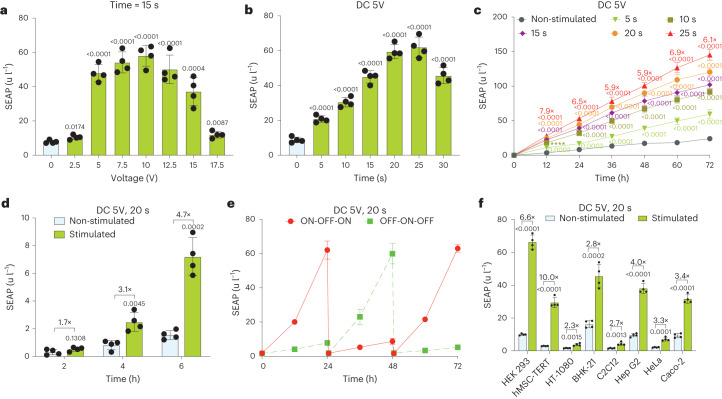
In vitro characterization of the DART system. **a**,**b**, SEAP levels 24 h after electrical stimulation in culture supernatants of HEK293 cells co-transfected with the DART constructs (pJH1003, pJH1004 and pJH1005). Stimulation for 15 s at the indicated voltages (**a**), or with 5 V DC for the indicated time periods (**b**). **c**, SEAP production kinetics over 72 h by engineered cells exposed to 5 V DC for the indicated periods of time. The induction factors were calculated between stimulated and non-stimulated group at 25 s. **d**, SEAP produced within 6 h by DART-engineered cells after exposure to 5 V DC for 20 s. The induction factors were calculated between the indicated groups. **e**, Reversibility of the DART switch. Engineered cells were alternately cultured for 24 h cycles in medium without electrostimulation (OFF) or treated with 5 V DC for 20 s (ON) and SEAP production was measured in the culture supernatants. Every 24 h, the culture medium was exchanged and the cell density was re-adjusted. **f**, SEAP produced by different mammalian cell lines transiently transfected with the DART constructs and stimulated with 5 V DC for 20 s. Non-stimulated cultures were used as controls. All data are mean ± s.d.; *n* = 4. *P* values were calculated between stimulated and non-induced control. Statistical designations with different colors refer to the corresponding data points with the same color (**c**). Source data

Profiling transgene expression levels following voltage-dependent DC electrostimulation for 15 s revealed similar expression levels between 5 and 12.5 V, whereas higher voltages generated higher ROS levels that decreased cell viability (Fig. 2a and Supplementary Fig. 5a). With the DC power set to 5 V, the expression level of the target gene could be precisely adjusted (Fig. 2b and Supplementary Fig. 5b,c) and distinct induction profiles were maintained over longer periods of time (Fig. 2c), during which correlating timelapse fluorescence microscopical analysis of enhanced green fluorescent protein showed no substantial leakiness of the DART system (Supplementary Fig. 5d–f and Supplementary Videos 2 and 3). Nevertheless, exposure times beyond 30 s significantly decreased both cell viability and SEAP expression (Supplementary Fig. 5g,h), likely as a result of gas bubbles and pH changes occurring at the electrodes^37^ (Supplementary Fig. 6a–e). The induction kinetics revealed significant levels of electrostimulated protein production in the culture supernatant within 4 h (Fig. 2d), which is consistent with the behavior of professional secretory cells^32^. Additionally, DC-powered transgene expression control was fully reversible, showing similar induction and repression profiles over several cycles of ON-to-OFF and OFF-to-ON switching (Fig. 2e).

To further assess the versatility of the DART system, we transiently transfected a set of mammalian cell lines (Fig. 2f). Despite variations in transfection efficiency^11^ and ROS sensitivity, resulting in different fold inductions and maximum expression levels, DART functionality was validated in all the tested cell lines, including human mesenchymal stem cell-derived cell line (hMSC-TERT), suggesting that DART will be compatible with a wide range of applications (Fig. 2f and Supplementary Fig. 6f). Taking into account the fold induction, basal and maximum expression levels, we selected HEK293 and hMSC-TERT cells for use in follow-up experiments (Fig. 2f).

### DART stimulation by off-the-shelf consumer batteries

**Fig. 3:**
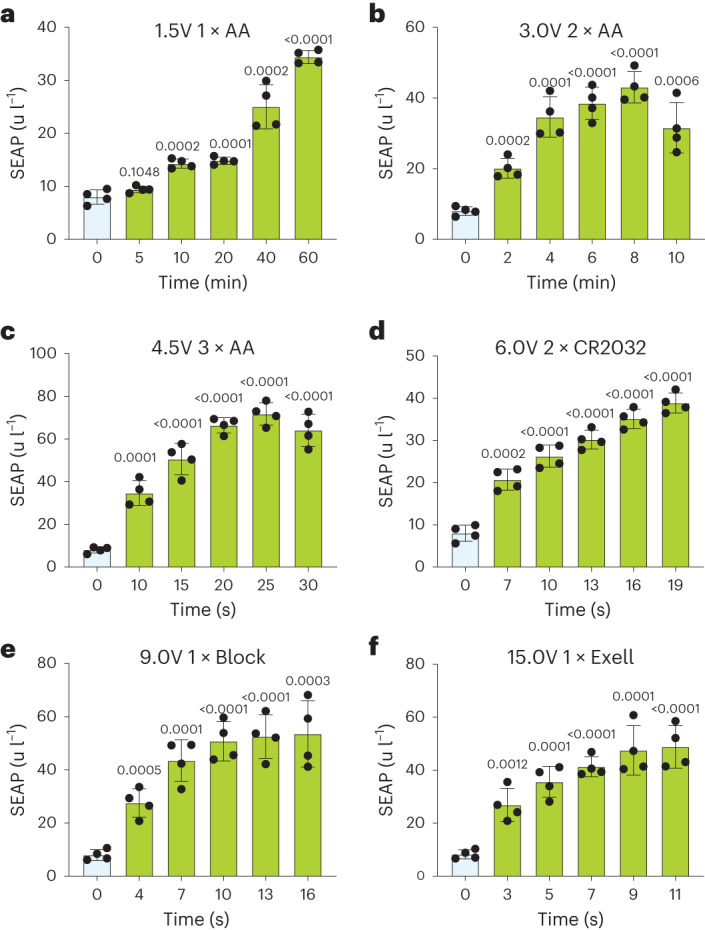
SEAP expression by DART-engineered cells stimulated for the indicated time periods with various DC supplies. **a**–**c**, One (**a**), two (**b**) and three (**c**) Duracell or Energizer alkaline 1.5 V AA batteries. **d**, Two Duracell or Energizer lithium button cells (CR2032) provide about 6 V. **e**, One Duracell or Energizer 9 V block alkaline battery. **f**, One A220/504 A Exell battery. SEAP levels were measured 24 h after stimulation. All data are mean ± s.d.; *n* = 4. *P* values were calculated between stimulated and non-induced control. Source data

**Extended Data Fig. 5:**
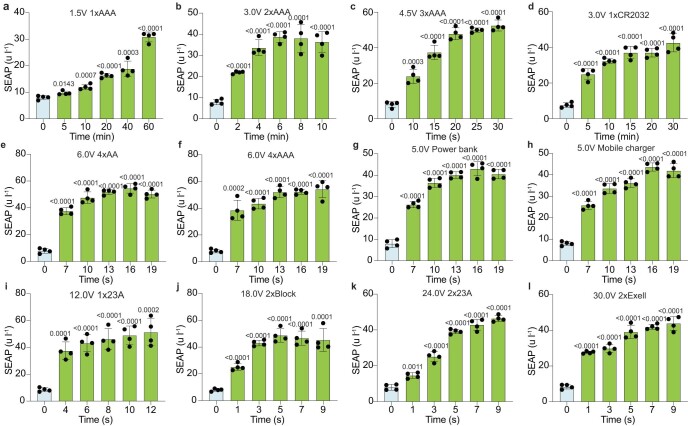
SEAP production by DART-engineered HEK-293 cells upon electrostimulation with different types of batteries. **a-c**, One (**a**), two (**b**) and three (**c**) Duracell or Energizer alkaline AAA batteries were used, each providing around 1.5 volts. **d**, One Duracell or Energizer lithium button cell CR2032 provides about 3 volts. **e-f**, Four Duracell or Energizer alkaline AA (**e**) and AAA (**f**) batteries provide around 6 volts. **g**, A Belkin power bank whose universal USB-A 2.4 A port delivers up to 5 volts. **h**, Apple and Huawei mobile phone chargers. The charger was directly plugged into mains electricity and the charger wires were cut for connection to the electrodes submerged in the culture medium. **i**, One Duracell or Energizer 12 volt 23 amp alkaline battery. **j**, Two Duracell or Energizer 9 volt block alkaline batteries. **k**, Two Duracell or Energizer 12 volt 23 A alkaline batteries. **l**, Two A220/504 A Exell batteries provide around 30 volts. SEAP levels in supernatants of culture medium were measured at 24 h after electrostimulation for the indicated time. Columns each represent the mean ± SD of the four indicated data points. *P* values were calculated between stimulated and non-stimulated group. Source data

To check the suitability of widely available DC power sources, as well as compatibility with portable and wearable electronic devices, we next examined electrostimulation of gene expression using standard off-the-shelf DC supplies such as alkaline batteries, button cells, mobile chargers and portable power banks. We first tested one, two and three 1.5 V AA and AAA batteries that provide 1.5, 3 and 4.5 V when connected in series, respectively. Although voltages <3 V required longer induction times of 2–60 min to produce sufficient ROS to trigger significant SEAP levels, stimulation with three AA or three AAA batteries for 10 s was sufficient to induce SEAP expression, which peaked at 25 s of stimulation (Fig. 3a–c and Extended Data Fig. 5a–c), showing induction profiles comparable to those generated by a 5 V power supply.

The 3 V CR2032 lithium button cell battery, which is widely used to drive wearable devices, triggered SEAP expression in up to 30 min, whereas a 6 V in-series setup of two CR2032s programmed peak SEAP expression levels in <20 s (Fig. 3d and Extended Data Fig. 5d). Furthermore, a 6 V in-series setup powered by four AA or four AAA batteries produced protein expression profiles comparable to those generated by the 2 × CR2032 configuration, corroborating the voltage-dependence of the electrogenetic gene switch independently of the type of power source (Extended Data Fig. 5e,f). This was further confirmed by using a mobile phone charger and a power bank that typically provide a 5 V output (Extended Data Fig. 5g,h).

We also tested higher-voltage batteries, including 9 V block, 12 V 23 A alkaline and 15 V Exell A220 504 A batteries in single (Fig. 3e,f and Extended Data Fig. 5i) or in-series tandem (Extended Data Fig. 5j–l) configurations. Measurements of SEAP expression confirmed an inverse correlation between voltage (1.5–30 V) and peak expression level times (5 s to 60 min) across all DC power sources (Fig. 3e,f and Extended Data Fig. 5i–l). Most notably, none of the tested battery configurations substantially impacted cell viability during the indicated stimulation times (Supplementary Figs. 7 and 8), confirming that the DC-powered electrogenetic interface is robust, safe and tunable across a wide range of voltages, battery types and stimulation times.

### Battery-powered insulin expression for the treatment of T1D

**Fig. 4:**
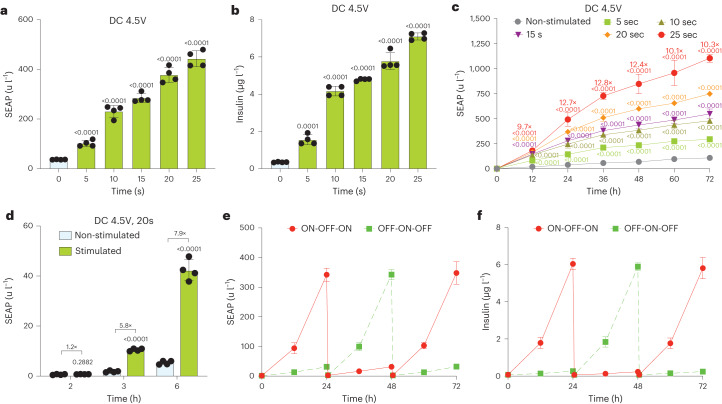
In vitro characterization of the monoclonal cell line. **a**,**b**, SEAP (**a**) and insulin (**b**) levels in culture supernatants of the monoclonal DC_INS_ cells stably containing the DART system 24 h after electrical stimulation. The voltage to stimulate the cells was provided by three AA batteries (4.5 V DC) for the indicated periods of time. **c**, SEAP production kinetics during 72 h after exposure of DC_INS_ cells to 4.5 V DC for the indicated time periods. The induction factors were calculated between non-stimulated and stimulated group at 25 s. **d**, SEAP produced during the first 2, 3 and 6 h by DC_INS_ cells non-stimulated or stimulated with 4.5 V DC for 20 s. The induction factors were calculated between the indicated groups. **e**,**f**, Reversibility of DC_INS_ cells expressing SEAP (**e**) and insulin (**f**). DC_INS_ cells were alternately cultured for 24-h cycles in medium treated with 5 V DC for 20 s (ON) or without electrostimulation (OFF). Culture supernatant samples were collected every 12 h for analysis of SEAP and insulin production. The culture medium was exchanged and the cell density was re-adjusted every 24 h. All data are means ± s.d.; *n* = 4. The *P* value indicates the significance of differences in the mean values; indicated group versus the non-stimulated group (**a**,**b**,**d**). The statistical designations with different colors refer to the corresponding data points with the same color (**c**). Source data

**Extended Data Fig. 6:**
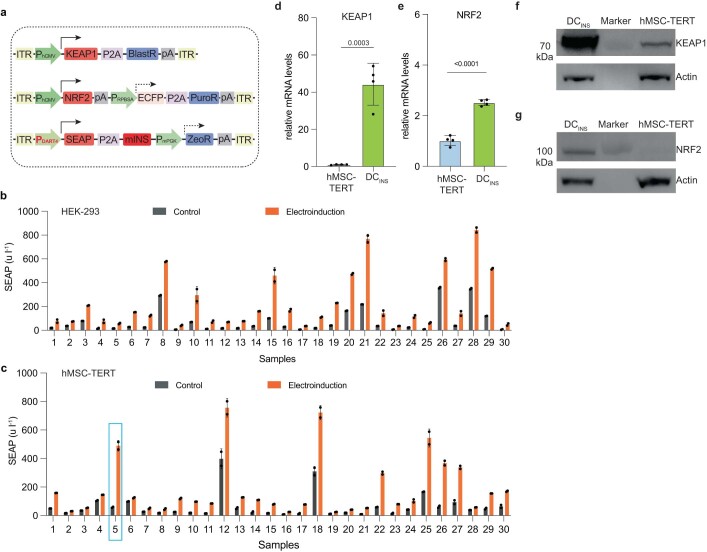
Generation and characterization of monoclonal cell lines. **a**, Design of DNA constructs used for the monoclonal cell lines. The DART system is based on the constitutive expression of KEAP1 (ITR-P_hCMV_-KEAP1-P2A-BlastR-pA-ITR, pJH1054) and NRF2 (ITR-P_hCMV_-NRF2-pA:P_RPBSA_-ECFP-P2A-PuroR-pA-ITR, pJH1101), and four tandem ARE (DART4)-controlled SEAP reporter followed by mouse insulin (mINS) (ITR-P_DART4_-SEAP-P2A-mINS: P_mPGK_-ZeoR-pA-ITR, pJH1169). All the constructs are flanked by inverted terminal repeats (ITR) for the recognition of SB100X-based Sleep Beauty transposase. **b**,**c**, Screening of monoclonal HEK-293 (**b**) and hMSC-TERT (**c**) cell lines. HEK-293 or hMSC-TERT cells were stably transfected with pJH1101, pJH1054 and pJH1169. Thirty monoclonal cell lines were randomly selected for profiling of SEAP expression at 24 hours after electrostimulation at 4.5 volts DC for 20 s. In (**c**), the blue rectangle indicates the best-in-class monoclonal cell line, DC_INS_, which was selected for follow-up experiments. The monoclonal cell lines were cultured and selected in medium supplemented with puromycin (2.5 μg/mL), blasticidin (10 μg/mL) and zeocin (100 μg/mL). Data in (**b**) and (**c**) are mean ± SD; n = 2; the SEAP values were normalized by cell numbers. **d-g**, Analysis of NRF2 and KEAP1 mRNA and protein levels in parental and DART-engineered cells. **d**,**e**, Relative mRNA levels of KEAP1 (**d**) and NRF2 (**e**) in parental cells (hMSC-TERT) and derived DC_INS_ cells, analyzed by quantitative real-time PCR. Columns each represent the mean ± SD of the four indicated data points. **f**,**g**, Western blot analysis of KEAP1 (**f**) and NRF2 (**g**) proteins in parental cells (hMSC-TERT) and derived DC_INS_ cells. The same amount of cell lysate was loaded on the gel and the housekeeping protein actin was used as a loading control. The experiments were repeated independently with similar results in **d**-**g**. *P* values were calculated between stimulated and non-stimulated group or indicated groups. Source data

As a challenging in vivo proof-of-principle, we chose to treat experimental type1 diabetes (T1D), because diabetes is a chronic disease that shows dramatically increasing prevalence globally and requires dynamically demanding management^45^. For DC-sensitive control of insulin production and release, we established stable HEK293 and hMSC-TERT cell lines engineered for constitutive expression of KEAP1 (ITR-P_hCMV_-KEAP1-P2A-BlastR-pA-ITR, pJH1054), NRF2 (ITR-P_hCMV_-NRF2-pA:P_RPBSA_-ECFP-P2A-PuroR-pA-ITR, pJH1101) and NRF2-dependent expression of insulin (ITR-P_DART4_-SEAP-P2A-mINS-pA:P_mPGK_-ZeoR-pA-ITR, pJH1169, P_DART4_, O_ARE4_-P_hCMVmin_) (Extended Data Fig. 6a). To maximize the dynamic range of insulin expression, we used the synthetic promoter variant P_DART4_, which contains four tandem ARE operator sites (Fig. 1f). Several monoclonal cell lines were profiled for DC-controlled insulin expression and the best-in-class hMSC-TERT cell clone DC_INS_ (Extended Data Fig. 6b,c), which showed improved electrostimulated insulin fold induction and release compared to transiently transfected isogenic cell populations (Extended Data Fig. 6c), was selected for treatment of experimental T1D. We confirmed by qPCR and western blotting that the DC_INS_ cell line had increased levels of KEAP1 and NRF2 transcripts and proteins when compared to the parental cell line (Extended Data Fig. 6d–g). Stimulation of DC_INS_ cells with 4.5 V DC from three AA batteries for exposure times between 5 s and 25 s could precisely regulate SEAP (Fig. 4a) and insulin (Fig. 4b) expression levels and distinct induction profiles were maintained over longer periods of time (Fig. 4c). Examination of the induction kinetics revealed that electrostimulated protein production reached a significant level in the culture supernatant within 3 h (Fig. 4d), which is faster than in the case of transiently transfected cells (Fig. 2d). Furthermore, the DC_INS_ cells exhibited excellent reversibility of SEAP (Fig. 4e) and insulin (Fig. 4f) expression in response to ON-OFF-ON or OFF-ON-OFF stimulation patterns at 24-h intervals.

**Fig. 5:**
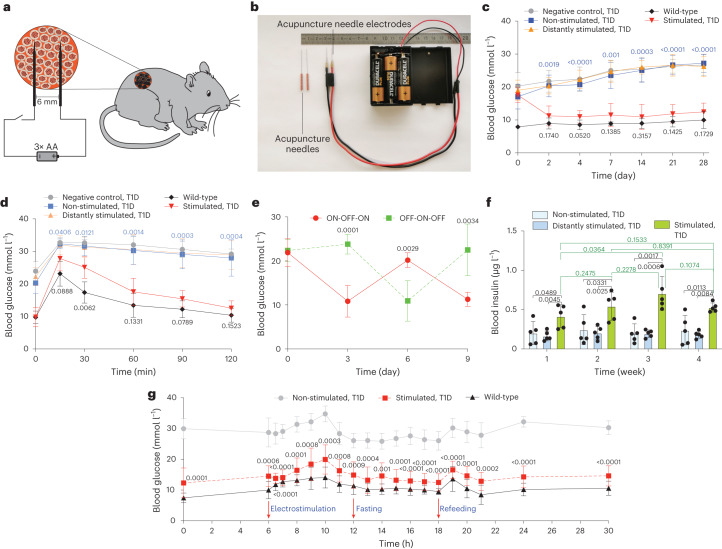
Adaptation and validation of the DART system to treat type1 diabetic mice. **a**, Scheme showing how encapsulated DART-engineered cells implanted in the back of mice are stimulated. **b**, Picture showing the customized acupuncture needles connected to the alkaline batteries. **c**, Fasting glycemia was recorded before implantation (day 0) and for four consecutive weeks after implantation in three groups of T1D mice with DC_INS_ cell implants, namely non-stimulated mice, mice stimulated for 10 s at the implantation site (stimulated) and mice with cell implants stimulated for 10 s with electrodes placed on their back 3 cm away from the implant site (distantly stimulated). Wild-type mice and T1D mice without implants were also used as controls. **d**, Intraperitoneal GTT was performed on mice 3 d after implantation of microencapsulated cells and after fasting for 8 h. **e**, Reversibility of DART-mediated glycemic control. Microencapsulated cells were percutaneously electrostimulated at the implantation site while reversing the ON-OFF/OFF-ON stimulation every third day, using 4.5 V DC for 10 s as ON. **f**, Blood insulin levels of non-stimulated and electrostimulated animals were profiled 1, 2, 3 and 4 weeks after implantation. Stimulated and distantly stimulated (3 cm away from the implant site) groups were treated with 4.5 V DC for 10 s per day (**c**,**f**). **g**, Blood glucose excursions on day 4 after implantation. Blood samples were collected at several time points for analysis of glucose in wild-type mice and T1D mice non-stimulated and stimulated with DC 4.5 V for 10 s. Time zero corresponded to midnight and electrostimulation was performed at 6:00. All data are mean ± s.d.; *n* = 5; the values were normalized to the wild-type group. The experiment was performed once in **g**. *P* values indicates the significance of differences in the mean values; stimulated group versus non-stimulated group (blue) or wild-type group (black) (**c**,**d**); stimulated group versus non-stimulated group (**g**); two groups versus each other (**e**); and stimulated group versus the indicated group (**f**). Source data

**Extended Data Fig. 7:**
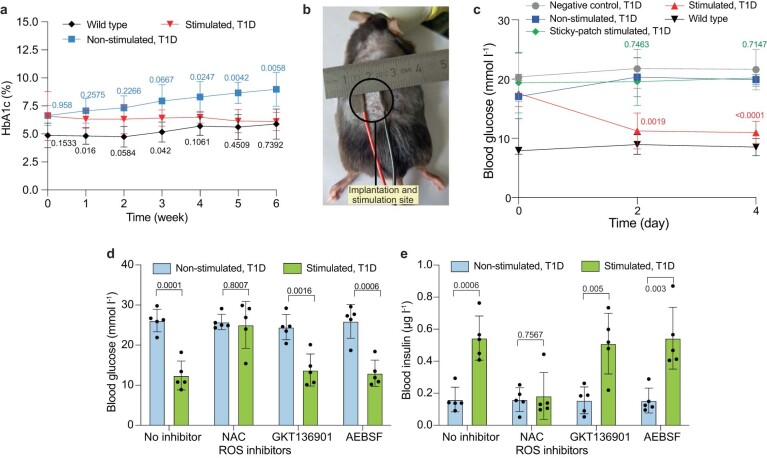
Profiling of glycated hemoglobin (HbA1c) and characterization of the ROS-mediated DART in mice. HbA1c levels in the blood of wild-type mice, and non-stimulated and electrostimulated T1D mice were measured weekly during six consecutive weeks after implantation of alginate-encapsulated DC_INS_ cells. **b,c**, Electrostimulation of mice with electrically conductive sticky electrodes. **b**, Picture of a mouse with conductive sticky electrodes attached to the implantation site at a distance of 6 mm (black circle). **c**, Fasting glycemia was recorded before implantation (day 0) and during four days post DC_INS_ cell implantation in three groups of T1D mice, namely mice stimulated with 4.5 volts DC for 10 s at the implantation site, with either sticky patches on the skin above the implantation site (Sticky-patch stimulated, green) or platinum acupuncture needles (Stimulated, red) and ’Non-stimulated‘ (blue) mice. Wild-type mice (black) and T1D mice without implants (Negative control, gray) were also used as controls. This experiment was not repeated. **d,e**, Administration of ROS scavenger and ROS-generating enzyme inhibitors to DART-implanted T1D mice. Blood glucose (**d**) and insulin (**e**) levels in non-stimulated and stimulated mice, subcutaneously injected with an ROS scavenger (NAC, 600 mg/kg) or inhibitors of ROS-generating enzymes (GKT136901, 50 mg/kg; AEBSF, 0.6 mg/kg) at the implantation site, 30 min before electrostimulation (DC 4.5 volts for 10 s). Blood samples were collected for analysis 4 days post implantation. Data points represent the mean ± SD; n = 5; the values were normalized to wild-type group. Statistical analysis was performed between: in (**a**), stimulated and non-stimulated groups (blue), and stimulated and wild-type groups (black); in (**c**), sticky-patch stimulated and non-stimulated (green), sticky-patch stimulated and stimulated (red); in (**d**) and (**e**), stimulated and non-stimulated group. Source data

**Extended Data Fig. 8:**
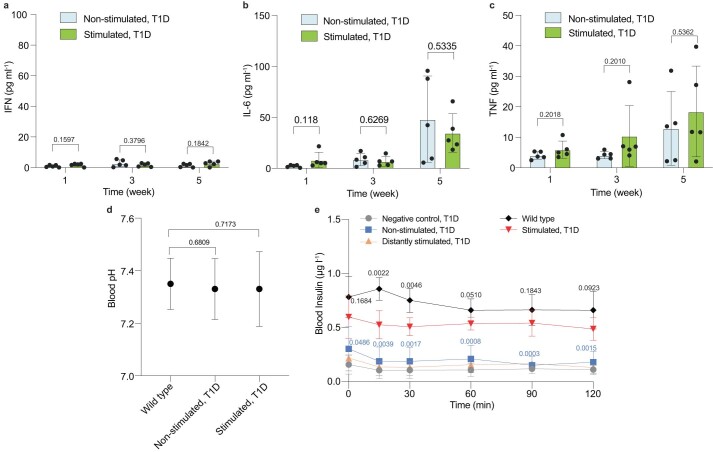
Profiling of inflammatory cytokines, blood pH and GTT insulin levels of T1D mice. **a-c**, IFN-γ (**a**), IL-6 (**b**) and TNF-α (**c**) levels were measured in the serum of non-stimulated and stimulated T1D mice, at the indicated time points after implantation of alginate-encapsulated DC_INS_ cells. **d**, Blood pH analysis. Blood samples were collected from three groups (wild-type, non-stimulated T1D and stimulated T1D mice) 3 min after electrostimulation with DC 4.5 volts for 10 s. **c**, The intraperitoneal glucose tolerance test **(**GTT) was performed after 8 h of fasting by administration of 1.5 g/kg aqueous D-glucose to mice on day 3 after implantation of microencapsulated cells. Blood samples were taken at the indicated time points. Data points represent the mean ± SD; in (**a**), (**b**), (**c**), (**e**), n = 5; in (**d**), n = 10; in (**d**) and (**e**), the values were normalized to wild-type group. In (**a**), (**b**), (**c**), statistical analysis was performed between stimulated and non-stimulated groups; in (**d**), statistical analysis was performed between wild-type and T1D group; in (**e**), statistical analysis was performed between stimulated and non-stimulated groups (blue), and stimulated and wild-type groups (black). Source data

**Extended Data Fig. 9:**
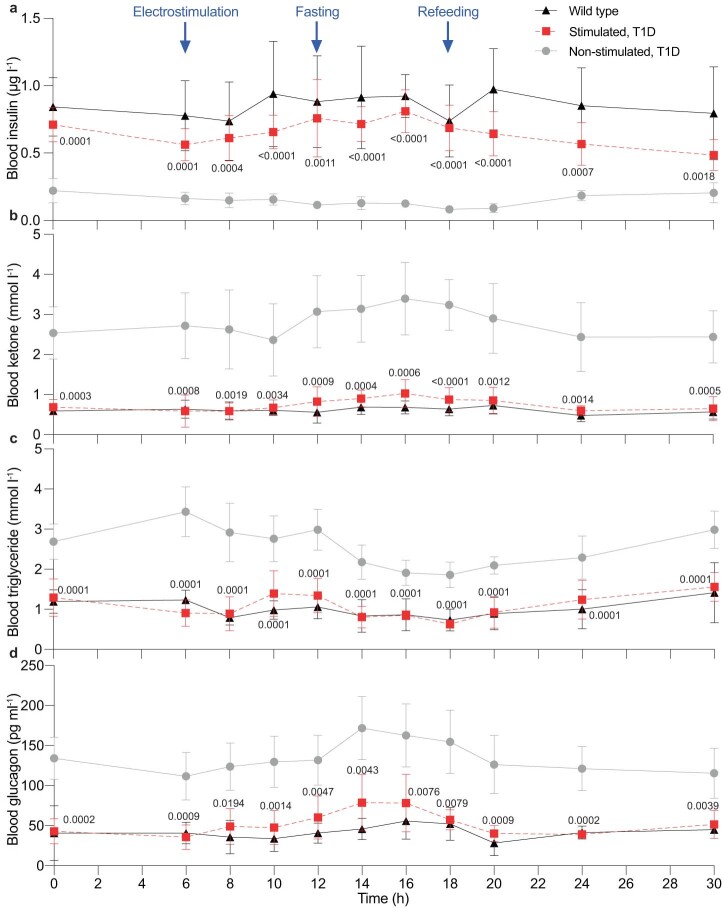
Profiling of blood insulin and biomarkers of insulin deficiency in serum of mice over a period of 30 hours. **a-d**, Insulin (**a**), ketone (**b**), triglyceride (**c**) and glucagon (**d**) levels were measured in the serum of wild-type, stimulated T1D and non-stimulated T1D mice on day 4 after implantation of alginate-encapsulated DC_INS_ cells. The stimulated animals were consecutively electrostimulated for three days at DC 4.5 volts for 10 s. On day 4, electrostimulation was performed at 6 a.m., and then the animals were fasted for 6 h. The blood samples were taken at the indicated time points. There is no notable difference between the electrostimulated and wild-type treatment groups at any time point. At 6 a.m. blood samples were collected immediately after electrostimulation and profiled for blood insulin and biomarkers of insulin deficiency. The corresponding profiles of blood levels of glucose are shown in Fig. 5g. Data points represent the mean ± SD, n = 5; the mice were monitored one time; the values were normalized to wild-type group. *P* values were calculated between stimulated and non-stimulated groups. Source data

As DC-electrostimulated DART-controlled insulin expression does not require any complex control electronics, we used a triple AA battery pack providing 4.5 V DC, wired via a simple manual ON/OFF power switch to two customized platinized acupuncture needles (Supplementary Fig. 9a–i and Supplementary Table 6) located 6 mm apart at the implantation site to stimulate DC_INS_ subcutaneously implanted on the back of type1 diabetic mice (Fig. 5a,b). Before implantation, we confirmed that the DC_INS_ cells microencapsulated in clinically licensed semi-permeable alginate showed precise time-dependent insulin release when stimulated with 4.5 V DC (Supplementary Fig. 9j). To confirm that insulin is produced and secreted in response to direct electrostimulation of implanted cells, we used a negative control group with similar dorsal implantation, but stimulated with acupuncture needles placed 3 cm away from the implant site. In the treated group, a single 4.5-V electrostimulation for 10 s per day attenuated fasting glycemia within 2 d and normoglycemia was restored over the whole treatment period of 4 weeks (Fig. 5c). In agreement with these results, glycated hemoglobin (HbA1c) levels were attenuated in electrostimulated T1D mice over 5 weeks of treatment, reaching similar levels to those of wild-type mice (Extended Data Fig. 7a). In contrast, when stimulating T1D mice with two electrically conductive sticky patches attached to the implantation site instead of the platinized acupuncture needles, the animals remained as hyperglycemic as non-stimulated T1D mice, indicating that this stimulation method cannot trigger insulin production from implanted DC_INS_ cells (Extended Data Fig. 7b,c). We also assessed how mice responded to the acupuncture needle stimulation when pretreated with NAC (ROS scavenger) or inhibitors of ROS-generating enzymes. In line with the in vitro results, only NAC abrogated the effect of electrostimulation, with mice showing blood glucose and insulin levels similar to those of non-stimulated mice (Extended Data Fig. 7d,e). Macroscopic assessment of the implantation site 4 weeks after transplantation showed no obvious differences between stimulated and non-stimulated mice or compared to non-transplanted wild-type mice (Supplementary Fig. 10a–c). To confirm that stimulation has no impact on surrounding tissues, we analyzed tissues adjacent to the electrode site of stimulated and non-stimulated mice according to ISO 10993-6 (ref. ^46^). We observed no material or electrostimulation-related cytotoxicity and no notable local-immune response around the implantation site (Supplementary Fig. 10d–g and Supplementary Table 7). There was no apparent histopathological difference between the electrode-containing non-stimulated and electrode-containing electrically stimulated groups (Supplementary Fig. 10d–g and Supplementary Table 7). Profiling of inflammatory mediators in serum of non-stimulated and electrostimulated groups also showed no significant differences (Extended Data Fig. 8a–c). Also, the short electrostimulation had no apparent effect on blood pH (Extended Data Fig. 8d). Glucose tolerance tests (GTTs) revealed that electrostimulated insulin production and release not only restored glucose homeostasis, but also attenuated postprandial glycemic excursion compared to negative control groups without any implant, or with non-stimulated implants, or with implants but distant electrostimulation (Fig. 5d and Extended Data Fig. 8e). Glycemic control was completely reversible when the electrostimulation status was switched from OFF to ON or from ON to OFF at 3-d intervals (Fig. 5e). Electrostimulated animals had significantly higher blood insulin levels than the control groups, confirming that DC-powered induction of insulin production and secretion indeed restored glucose homeostasis in type 1 diabetic mice (Fig. 5f). Furthermore, we profiled the blood glucose levels several times a day, during 24 h after electrostimulation with DC 4.5 V for 10 s and did not observe any hypo- or hyper-glycemic episodes (Fig. 5g). In agreement with this finding, the time-course analysis of other biomarkers of insulin deficiency, namely insulin, ketones, triglycerides and glucagon, revealed no significant differences between electrostimulated T1D mice and wild-type animals, whereas those biomarkers were significantly lower (insulin) and higher (ketones, triglycerides and glucagon) in non-stimulated T1D mice, respectively (Extended Data Fig. 9).

### Tunability of DART in vivo

**Fig. 6:**
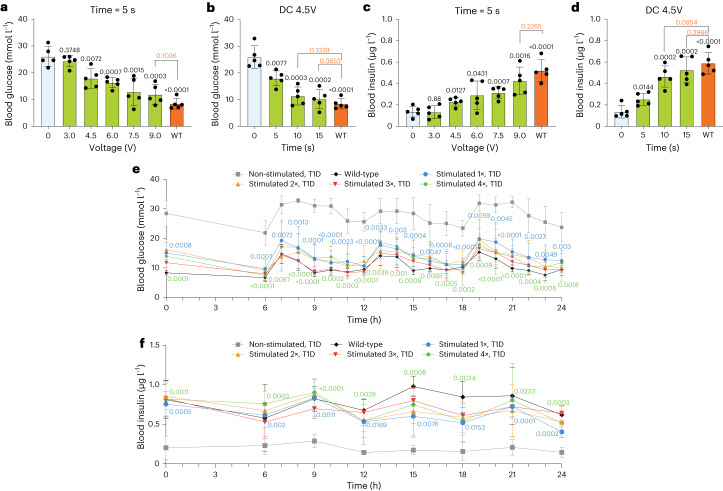
Evaluation of the tunability of DART system in T1D mice. **a**–**d**, Blood glucose (**a**,**b**) and insulin (**c**,**d**) levels of T1D mice with DC_INS_ cell implants electrostimulated with different voltages (3, 4.5, 6, 7.5 and 9 V) for 5 s (**a**,**c**) and for different periods of time (0, 5, 10 and 15 s) at 4.5 V (**b**,**d**). The voltages were applied with two to five AA batteries and one 9 V block battery, respectively. T1D and wild-type mice without any treatment were used as controls. The blood samples were taken after 6 h of fasting. The corresponding blood profiles of biomarkers of insulin deficiency, ketones, triglycerides and glucagon are shown in Extended Data Fig. 10a–f, Time-series blood glucose (**e**) and insulin (**f**) monitoring on day 4 after implantation in T1D mice non-stimulated or stimulated once, twice, three or four times per day with DC 4.5 V for 10 s according to the scheme in Extended Data Fig. 10g. The corresponding blood profiles of biomarkers of insulin deficiency, ketones, triglycerides and glucagon are shown in Extended Data Fig. 10h–j. All data are mean ± s.d.; *n* = 5; the experiment was performed once and values were normalized to the wild-type group. The *P* value indicates the significance of differences in the mean values. Statistical designations with different colors refer to the corresponding data points with the same color (**e**,**f**). Source data

**Extended Data Fig. 10:**
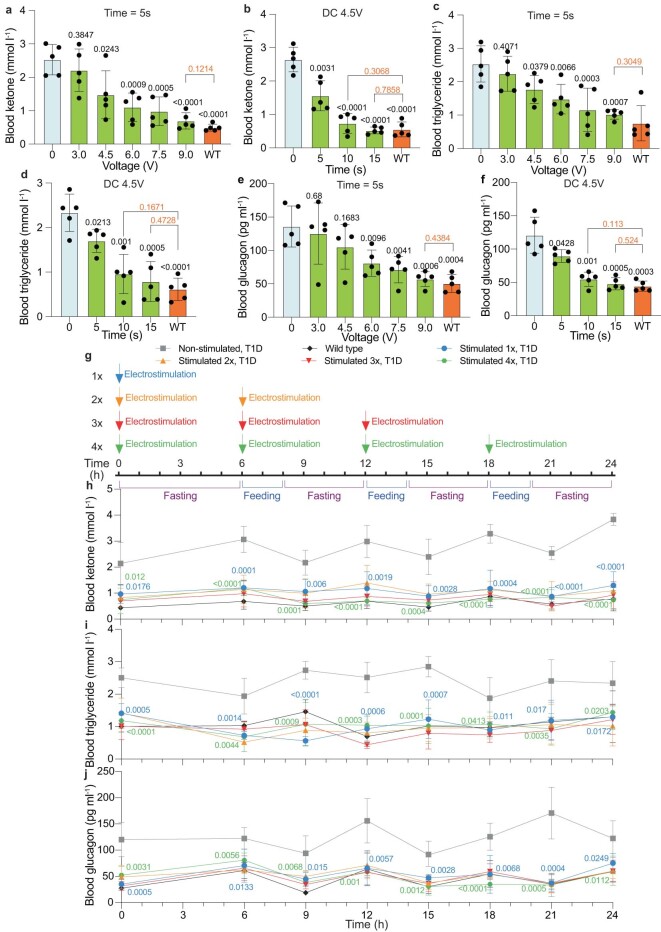
Profiling of biomarkers of insulin deficiency in serum of mice. **a-f**, the T1D mice were electrostimulated using various voltages for different periods of time. **a-f**, Ketone (**a,b**), triglyceride (**c,d**) and glucagon (**e,f**) levels were measured in the serum of wild-type, stimulated and non-stimulated T1D mice on day 4 after implantation of alginate-encapsulated DC_INS_ cells. The stimulated groups were consecutively electrostimulated for three days at the indicated voltages for the indicated time periods. Two, three, four and five AA batteries provided DC 3.0, 4.5, 6.0, 7.5 volts, respectively. One 9 V block provided DC 9.0 volts. The wild-type samples were from two different groups of mice without any treatment. The blood samples were taken before fasting on day 4. The corresponding profiles of blood levels of glucose and insulin are shown in Fig. 6a-d. g-j, profiling of biomarkers of insulin deficiency in serum of mice with different induction times. **g**, Schedule of electrostimulation with different frequencies and the fasting-feeding cycle for all mice throughout the experiment. **h-j**, Ketone (**h**), triglyceride (**i**) and glucagon (**j**) levels were measured in the serum of wild-type, stimulated T1D and non-stimulated T1D mice on day 4 after implantation of alginate-encapsulated DC_INS_ cells. The stimulated groups were consecutively electrostimulated for three days at DC 4.5 volts for 10 s with the indicated induction frequencies. The blood samples were taken on day 4 at the indicated time points. The corresponding profiles of blood levels of glucose and insulin are shown in Fig. 6e,f, respectively. Statistical analysis was performed between non-stimulated and stimulated groups or indicated groups. The statistical designations with different colors in (**h**)-(**j**) refer to the corresponding data points with the same color. Data points represent the mean ± SD; n = 5; the experiment was performed one time; the values were normalized to wild-type group. Source data

To assess the tunability of the DART system in vivo, we stimulated T1D mice during different time periods (between 5 to 15 s) using two to five AA batteries and one 9 V block battery as power sources, providing 3, 4.5, 6, 7.5 and 9 V of DC, respectively. We observed voltage-dependent as well as time-dependent blood glucose (Fig. 6a,b) and blood insulin (Fig. 6c,d) tunability as well as concomitant fine-tuning of other biomarkers of insulin deficiency, ketones, triglycerides and glucagon (Extended Data Fig. 10a–f), showing that metabolic homeostasis could be restored by applying 4.5 V electrostimulation for 10 s or more (Fig. 6b,d) and confirming the in vitro tunability of DART-transgenic cells (Figs. 2b,c and 4a–c, Extended Data Figs. 3g,h and 7j and Supplementary Fig. 5c).

Finally, we assessed how the implanted DART system responds to up to four electrostimulations per day, to mimic the need for multiple daily insulin injections in some patients with T1D. To mimic the pattern in humans, we scheduled repeated fasting–feeding cycles for the mice throughout the day (Extended Data Fig. 10g). In all electrostimulated groups, the glucose and insulin levels fluctuated around the wild-type levels and all showed significant differences from the non-stimulated group at each time point (Fig. 6e,f). To make a comprehensive assessment of the diabetic status of all groups, we took blood samples every 3 h in the daytime and tested for ketone bodies, triglycerides and glucagon. The results indicated that all the electrostimulation schedules, from one to four per day, significantly improved the glycemic state of diabetic mice (Extended Data Fig. 10h–j).

## Discussion

DART provides a reversible and tunable electrogenetic interface operated by simple, readily available low-voltage DC sources, such as batteries that are widely used to power portable or wearable electronic devices^18,47^. Notably, DART requires very little power and overall energy to control target gene expression. As a single electrical stimulation using two electrodes 6 mm apart for only 10 s is sufficient to trigger production and release of sufficient daily insulin, we estimate the electrical DC power required for a daily insulin shot to be around 0.06 W, which would enable a simple 4.5 V triple AA battery pack to operate for more than 5 years, while providing a daily therapeutic dose. In principle, operating times could be further optimized by decreasing the resistance by reducing the electrode separation, or as we have shown, by using higher-voltage and higher-capacity battery packs to achieve even shorter induction times. Furthermore, control of DART requires only a simple manual electrical ON/OFF switch. Also, as DART can directly stimulate engineered cells that are microencapsulated in US FDA-approved alginate microcontainers that are clinically licensed and validated for human islet transplantation^48^ via WHO-approved and US FDA-licensed acupuncture needles^49^, the remote control is simple and does not require the use of complex, failure-prone bioelectronic implants, which are particularly challenging to operate in a tissue environment^32^. Indeed, our first electrogenetic cell implant device relied on the sensitization of the cells to electrical fields by coexpression of two ion channels linked to endogenous signaling cascades and encapsulation of the cells in a complex wireless-powered bioelectronic implant, activated by AC at high voltage with extended stimulation times^32^. In contrast, the DART technology capitalizes on intracellular ROS sensors that sensitize alginate-encapsulated cells to direct battery-powered low-voltage DC stimulation within seconds via two simple acupuncture needles and without the need to use or implant any electronics. In fact, electrostimulation by acupuncture needles is already standard practice in traditional Chinese medicine for the treatment of inflammation, chronic pain, muscle spasm and neurological disorders^50,51^, representing a therapeutic modality that is approved by the WHO^49^ and practiced on a worldwide basis. As the DART system does not need hours at higher voltages but only seconds at lower voltages to actuate transgene expression, it has higher energy efficiency and safety. Thus, we believe rapid, electronics-free direct battery-powered low-voltage DC control of therapeutic transgenes in human cells is a leap forward, representing the missing link that will enable wearables to control genes in the not-so-distant future.

On the molecular side of the electrogenetic interface, DART taps into the ubiquitous KEAP1/NRF2-mediated sensing of ROS. ROS production is a part of the cellular respiratory process^34,52^, but we found that intrinsic levels of ROS production and the KEAP1/NRF2-mediated ROS management system do not interfere with DART and do not substantially induce DART-specific promoters. Instead, human cells need to be sensitized to electro-inducible ROS production by ectopic expression of native KEAP1 and NRF2, which reroute ROS sensing to synthetic P_DART_ promoters driving biopharmaceutical production. As with other synthetic transcription-control modalities, activation of endogenous genes cannot be ruled out completely^7^; however, comparative deep-sequencing analysis suggested that any such effect is minimal. Also, mass spectroscopic analysis of the culture medium showed no substantial difference between non-stimulated and electrostimulated cell cultures, indicating that DART activation does not cause sufficient electrolysis in the culture medium to perturb cellular systems. Furthermore, DART is exclusively composed of endogenous components, involving simple ectopic expression of native endogenous factors KEAP1 and NRF1 and target promoters assembled by fusing native tandem ARE elements to a minimal promotor box, which should minimize risks that can be associated with cell engineering, such as neoplastic transformation.

Qualitatively, electrostimulated ROS production is triggered by the creation of potentially hazardous chlorine ions and chlorine gas as well as protons and hydrogen gas at the anode and cathode, respectively; however, we found that electrostimulation had no negative impact either on the viability or the transcriptome of the cells or on the medium composition, presumably due to the low voltage and short induction times of only a few seconds. Indeed, a single daily electrostimulation of implanted engineered cells at 4.5 V for 10 s triggered production and release of sufficient insulin to restore normoglycemia in experimental T1D, exhibiting comparable efficacy to long-acting insulin therapies that can maintain fairly stable blood-sugar levels for 24 h^18,22,32^. DART control also provided sufficient insulin to rapidly attenuate postprandial glycemic excursion, as shown by GTTs. In addition, DART control was reversible and also finely tunable by varying the voltage and/or electroinduction period. Notably, we also evaluated several biomarkers of insulin deficiency in animals treated with the DART system using different batteries, different induction periods and different frequencies. The improvements of glucose and insulin levels confirm that DART is an efficient system. Furthermore, the improvement of ketone levels in diabetic mice would also reduce the risk of diabetic ketoacidosis.

While we chose DART-controlled insulin production for proof-of-concept validation, it should be straightforward to link DART control to the in situ production and dosing of a wide range of biopharmaceuticals. We believe simple electrogenetic interfaces such as DART that functionally interconnect analog biological systems with digital electronic devices hold great promise for a variety of future gene- and cell-based therapies, including closed-loop genetic interventions, real-time dosing and global telemetric monitoring by medical staff or algorithms.

## Methods

### Key plasmids used in this study

Construction details for all vectors are provided in Supplementary Table 1. Key plasmids included (1) a constitutive KEAP1 expression vector (pJH1004, P_hCMV_-KEAP1-pA) and the corresponding vector containing inverted terminal repeats (ITRs) of Sleeping Beauty (SB) transposase (pJH1054, ITR-P_hCMV_-KEAP1-P2A-BlastR-pA-ITR) for stable cell line generation; (2) a constitutive NRF2 expression vector (pJH1003, P_hCMV_-NRF2-pA and pJH1101, ITR-P_hCMV_-NRF2-pA: P_RPBSA_-ECFP-P2A-PuroR-pA-ITR); and (3) NRF2-dependent synthetic promoters containing ARE operator sites fused to a minimal promoter (pJH1005, P_DART_-SEAP-pA and pJH1169, ITR-P_DART4_-SEAP-P2A-mINS:P_mPGK_-ZeoR-pA-ITR).

### Cell culture and transfection

Human embryonic kidney cells (HEK293, ATCC, CRL-11268), human fibrosarcoma cells (HT-1080, ATCC, CCL-121), human cervical adenocarcinoma cells (HeLa, ATCC, CCL-2), human telomerase-immortalized mesenchymal stem cells (hMSC-TERT^53^, RRID:CVCL_Z015), human liver cancer cell line (Hep G2, ATCC, CRL-11997), human colorectal adenocarcinoma cell line (Caco-2, ATCC, HTB-37), mouse myoblast cell line (C2C12, ATCC, CRL-1772), baby hamster kidney cells (BHK-21, ATCC, CCL-10) and Chinese hamster ovary cells (CHO-K1, ATCC, CCL-61) were cultivated in Dulbecco’s modified Eagle’s medium (DMEM, cat. no. 52100-39, Thermo Fisher Scientific) supplemented with 100 mM proline (CHO-K1 only), 10% fetal bovine serum (FBS, cat. no. F7524, lot no. 022M3395, Sigma-Aldrich) and 1% (v/v) streptomycin/penicillin (cat. no. L0022, Biowest) and were grown at 37 °C in a humidified atmosphere containing 5% CO_2_. For transfection, 50,000 cells (CellDrop BF Brightfield Cell Counter, DeNovix) were seeded per well on a 24-well plate (cat. no. 3524, Corning Life Sciences), cultivated for 12 h and transfected by addition of 50 µl of a mixture containing 1.6 µg polyethyleneimine (PEI MAX, MW 40,000, 1 μg μl^−1^ in ddH_2_O, cat. no. 24765-2, Polysciences) and 0.5 µg plasmid DNA (equimolar concentrations for plasmid mixtures). After 8 h, the mixture was replaced with standard cultivation medium (700 µl).

### Monoclonal cell line design

A total of 1.5 × 10^5^ HEK293 or hMSC-TERT cells were co-transfected with pJH1101 (200 ng), pJH1054 (550 ng), pJH1169 (400 ng) and pJH42 (P_hCMV_-SB100X-pA) encoding constitutive expression of a hyperactive SB transposase^54^ (200 ng). After clonal expansion, the monoclonal cell lines were screened by electrostimulation and the best-in-class cell line DC_INS_ was selected for in vitro and animal studies.

### Effect of electrostimulation on viable cell growth and productivity

The 2.5 × 10^6^ HEK293 cells were seeded in a 10-cm diameter dish (Greiner Bio-one, cat. no. 664160) for 24 h before transfection with 30 μg pJH3 (P_hCMV_-SEAP-pA). The next day, the cells were resuspended and evenly reseeded into two 24-well plates. The treatment groups were stimulated at 5 V DC for different periods of time. Samples were collected at successive time points for 48 h to quantify viable cell count and SEAP production.

### Chemical ROS modulation

ROS-modulating compounds were provided 30 min before performing electrostimulation for both in vitro and in vivo experiments, at the concentrations indicated in the figure legends. All compounds used were from Sigma-Aldrich, namely NAC^40^ (cat. no. A7250-50G), GKT136901 (cat. no. 5340320001), AEBSF (cat. no. SBR00015-1ML), ML171 (cat. no. 492002-10MG) and VAS2870 (cat. no. SML0273-25MG)^41^.

### Analytical assays

#### Cell viability

Cells were incubated for 2 h with resazurin (50 μg ml^−1^, cat. no. R7017, Sigma-Aldrich) before recording the fluorescence at 540/590 nm (Tecan Infinite 200 PRO plate reader, Tecan Group AG)^32^.

#### SEAP quantification

SEAP levels were profiled in cell culture supernatants using a colorimetric assay. A total of 100 µl 2× SEAP assay buffer (20 mM homoarginine, 1 mM MgCl_2_ and 21% diethanolamine, pH 9.8) was mixed with 80 µl heat-inactivated (30 min at 65 °C) culture supernatant. After the addition of 20 µl substrate (120 mM p-nitrophenyl phosphate; cat. no. AC128860100, Thermo Fisher Scientific), the absorbance was recorded for 30 min at 405 nm and 37 °C (Tecan Infinite 200 PRO) and SEAP levels were determined as described previously^55^.

#### ROS quantification

Electrostimulated or chemically induced cells were washed with 300 µl phosphate-buffered saline (PBS, cat. no. 14190-094, Thermo Fisher Scientific), incubated for 45 min in 500 µl FBS-free DMEM containing 25 µmol l^−1^ 2′,7′-dichlorofluorescein diacetate (cat. no. D6883, Sigma-Aldrich) and washed again with 300 µl PBS and then ROS levels were quantified by fluorescence assay^56^ (485/535 nm, Tecan Infinite 200 PRO).

#### Insulin

Insulin was quantified by an ELISA kit (cat. no. 10-1247-01, Mercordia).

#### Glucose

Blood glucose was quantified using the clinically licensed ContourNext test strips and ContourNext ONE reader (Ascensia Diabetes Care)^57^.

#### Electrospray ionization mass spectrometry

The culture supernatants of DART-transgenic cells electrostimulated at 10 V DC for 30 s were directly analyzed by electrospray ionization mass spectrometry using non-stimulated isogenic cell cultures as negative controls. The samples were directly injected at 0.3 ml min^−1^ with an ultra-performance liquid chromatography device (UltiMate 3000, Thermo Fisher Scientific) and the electrospray ionization mass spectrometry spectra were recorded in positive and negative ion polarity modes using a maXis 4 G high-resolution mass spectrometer (Bruker) equipped with an electrospray ionization source set to 200 °C capillary temperature and 4.5 kV spray voltage.

#### Serum inflammatory cytokines

Mouse interferon (IFN)-γ, interleukin (IL)-6 and tumor necrosis factor (TNF)-α levels were quantified using IFN-γ (cat. no. BMS606-2), IL-6 (cat. no. BMS603HS) and TNF-α (cat. no. BMS607HS) mouse ELISA kits (all from Thermo Fisher), respectively.

#### Cl_2_ gas measurement

Chlorine gas dissolved in the culture medium was analyzed using test strips (DPD-1, cat. no. 486637), which were quantified by a photometer (eXact EZ Photometer, cat. no. 486205) (all from ITS Europe).

#### Medium and blood pH measurement

Medium pH was measured using high-accuracy pH test paper (cat. no. D-52348, MACHEREY-NAGEL) and blood pH was measured by a photometer (eXact EZ Photometer, cat. no. 486205, ITS Europe) with pH strips (cat. no. 486639, ITS Europe).

#### Western blot

Exponentially growing cells were lysed in ice-cold RIPA buffer (150 mM NaCl, 50 mM Tris-HCl 8.0, 1% Nodidet P-40 (NP40), 0.5% sodium deoxycholate, 0.1% SDS, 1 mM sodium orthovanadate, 1 mM NaF and protease inhibitors; Roche), during 30 min at 4 °C with continuous agitation, followed by spinning at 15,000 *g* for 20 min at 4 °C. The supernatants were transferred to fresh tubes on ice. The total protein concentration was quantified with a Pierce BCA Protein Assay kit (cat. no. 23225, Thermo Fisher). Then the samples were boiled in 2× Laemmli buffer at 95 °C for 5 min. Then, 20 μg of sample was resolved by SDS–PAGE and transferred to polyvinylidene fluoride membrane (cat. no. 88518, Thermo Fisher) in transfer buffer (25 mM Tris, 190 mM glycine and 20% methanol). After transfer, membranes were blocked with 5% nonfat milk for 1 h at room temperature and incubated with KEAP1 (cat. no. ab227828, Abcam; 1:5,000 dilution) and NRF2 (cat. no. ab137550, Abcam, 1:5,000 dilution) primary antibodies overnight at 4 °C, followed by incubation with secondary antibody (anti-rabbit IgG HRP Linked Whole antibody, cat. no. GENA934-1ML, Sigma-Aldrich, 1:10,000 dilution). Blots were visualized after adding the chemiluminescent substrate (Pierce ECL Western Blotting Substrate, cat. no. 32106, Thermo Fisher) with an chemiluminescence detection system (FusionPulse TS, cat. no. 121172301, v.5.12a). Mouse anti-β-actin (Sigma, cat. no. A2228, 1:5,000 dilution) and sheep anti-mouse IgG (Sigma, cat. no. GENA931V, 1:10,000 dilution) were used as a control.

#### Quantitative PCR assay

The messenger RNA samples from cultured cells were extracted using a Quick-RNA Miniprep kit (Zymo Research, cat. no. R1054) and quantified with a NanoDrop 2000 (Thermo Fisher). The complementary DNA library was constructed using a High-Capacity cDNA Reverse Transcription kit (Applied Biosystems, cat. no. 4368814). The qPCR analysis using SsoAdvanced Universal SYBR Green Supermix (Bio-Rad, cat. no. 1725271) was performed by QuantStudio 3 (Thermo Fisher). The primers used for *KEAP1*, *NRF2* and housekeeping genes are listed in Supplementary Table 8.

#### HbA1c assay

Serum HbA1c levels were quantified using a Mouse HbA1c Assay kit (cat. no. 80310, CrystalChemA).

#### Ketone, triglyceride and glucagon assays

Serum levels were quantified using ketone body (cat. no. MAK134-1KT, Sigma-Aldrich), triglyceride (ab65336, Abcam) and glucagon (cat. no. 10-1271-01, Mercodia) ELISA assay kits, respectively.

### RNA sample preparation and sequencing

HEK293 cells were seeded overnight, electrostimulated at DC 5 V during 10 or 25 s and 8 h after were collected for total RNA extraction using a Quick-RNA Miniprep kit (Zymo Research, cat. no. R1054). RNA quality was assayed by a BioAnalyzer (Agilent Technologies). The libraries were prepared with the Illumina Truseq stranded Total RNA Library PrepKit (Illumina). Each library was sequenced using an HiSeq 2500 system (Illumina), resulting in about 30 million single-end 81-mer reads located near the 3′ end of the mRNA per sample.

### RNA-seq data processing

Sequencing data were demultiplexed and primarily analyzed using a Snakemake workflow^58^, consisting of Trimmomatic (v.0.35), alignment to the GRCh38 genome with HISAT2 (v.2.1.0), SAMtools (v.1.9) to sort and index the alignment BAM files and featureCounts from Subread package (v.2.0.1) to count reads in the gene ranges, using human Ensembl annotation v.105. The count vectors for all samples were combined into a table, which was then subjected to the secondary analysis in R. The quality control and sample consistency were checked with principal-component analysis using R package PCATools. The count table was processed in the secondary (statistical) analysis with R scripts using edgeR (v.3.32)^59^, in particular, a binomial generalized log-linear model with contrast tests. It resulted in lists of genes ranked for differential expression by *P* value and used a Benjamini–Hochberg-adjusted *P* value as the estimate of the false discovery rate.

### Electrochemical deposition of Pt-PEDOT:PSS on acupuncture needles

Poly(3,4-ethylenedioxythiophene) polystyrene sulfonate (PEDOT:PSS) coating was conducted by anode deposition^60^ using an electrochemical workstation (CHI760E, serial no. E1174, v.20.4.0.0). The process was performed in 40 ml aqueous solution (0.1 M KCl and 5 ml PEDOT:PSS solution (1% w/w in dimethylsulfoxide)) at 3.0 V DC. An aqueous solution containing 5 mM H_2_PtCl_6_, 1 mM urea and 0.1 M H_2_SO_4_ was used for electrodeposition. Pt wire and PEDOT:PSS-coated acupuncture needles (PEDOT:PSS/SS) were used as the counter and working electrodes, respectively. DC electrodeposition was performed using a potentiostat (CHI760E) set to an optimized current density of −20 mA cm^−2^ for 10 min^61^. After electrodeposition, the Pt-PEDOT:PSS/SS electrodes were washed with Millipore water and dried at 90 °C for 6 h. The morphology of the probes was observed under a scanning electron microscope (FEI Sirion 400 NC) at an acceleration voltage of 5.0 kV. A CHI760E electrochemical analyzer controlled by CHI software was used to record the electrochemical current.

### Battery testing

Three tandem AA batteries (IEC name LR06; Energizer, cat. no. E300173103) were connected to a battery tester with a potentiostat (CHI760E). Galvanostatic discharge was measured by the chronopotentiometry method with the indicated anode current.

### Statistics and reproducibility

The data presentation, sample size of biological replicates (*n*), statistical analysis and significance of differences are shown in the figures. All in vitro experiments were reproduced at least twice, unless otherwise stated. For the mouse experiments, biological replicates (*n* = 5 mice) were used, unless otherwise stated. The details are described in each figure legend. To determine the statistical significance of differences in the case of multiple comparisons we used GraphPad Prism 8 (v.9.2.0, GraphPad Software) or Microsoft Excel (v.16.51, Microsoft) and a two-tailed, unpaired, Student’s *t*-test and one-way analysis of variance.

### Video filming

Supplementary Video 1 was filmed in an incubator at 37 °C and 5% CO_2_ using a Huawei P30 mobile phone. Supplementary Videos 2 and 3 were recorded on a Zeiss LSM 980 Airyscan microscopic system. The movies were further processed by Shotcut (v.21.09.20) and HandBrake (v.1.4.2) software.

### Electrostimulation

For in vitro electrostimulation we cultivated electrosensitive cells in standard 24-well plates with 700 µl culture medium, which allow the immersion of two platinum electrodes with 6-mm spacing fixed on a customized lid. The lid of a 24-well plate (cat. no. 3524, Corning Life Sciences) was glued with breadboards (cat. no. H25PR500, Reichelt Elektronik) and platinum wires (cat. no. HXA 050, Cooksongold; Supplementary Fig. 1a,b). Electrical power was applied with the indicated parameters and periods using a DC power supply (KD3005P, KORAD) or various battery packs. Square AC pulses were generated by an HP3245A Universal Source function generator (cat. no. 3245A, Hewlett Packard) connected to a linear amplifier P200 (cat. no. P200, FLC Electronics). The AC parameters used are indicated in the figure legends. The DC and AC voltages and currents were confirmed by connecting a CHI760E potentiostat and a digital oscilloscope (cat. no. DS1052E, Soochow). For in vivo electrostimulation of subcutaneously implanted microencapsulated cells, we used three AA batteries (IEC name LR06; Energizer, cat. no. E300173103) as the DC power source and sterile tip-platinized acupuncture needles or customized platinum electrodes (cat. no. 1503030, Wandrey) with 6-mm spacing as electrodes. Then, 1 ml fresh DMEM medium was injected at the implantation site before electroinduction.

#### Other DC power sources

In addition to AA batteries, we also used AAA batteries (IEC-LR03, Energizer, cat. no. E303271700), 9 V blocks (IEC-6LR61, Conrad, cat. no. CE-650900), button cells (IEC-CR2032, Energizer, cat. no. E303272400), 12 V 23 A alkaline batteries (IEC-8LR23, Energizer, cat. no. E301536201), A220/504 A Exell batteries (cat. no. 4331974451, Exell), a Belkin power bank (cat. no. 13350487) and Apple and Huawei USB-C mobile phone chargers for in vitro or in vivo experiments.

#### Electrochemical analysis

The electrodes were characterized by cyclic voltammetry. Calibration was performed in 10 mM potassium hexacyanoferrate (III) (K_3_Fe(CN)_6_) solution. The experiment was conducted with a CHI760E potentiostat using two platinum electrodes as working and counter electrodes and an Ag/AgCl reference electrode (CHI111P, IJ Cambria Scientific). Cyclic voltammograms were run between −0.3 and 0.6 V for calibration of the electrode and from −5 to 5 V for measurement at a scan rate of 10 mV s^−1^. The three-electrode system was used for ROS generation and SEAP induction with transient DART-engineered cells.

### Microencapsulation and implantation of electrosensitive DC_INS_ cells

To protect human DC_INS_ cells from the mouse immune system while enabling free diffusion of nutrients and therapeutic proteins, we used clinical trial-validated US FDA-licensed alginate-based encapsulation technology^48^. DC_INS_ were microencapsulated into coherent alginate-poly(l-lysine)-alginate microcapsules of 400 µm in diameter by mixing 1.0 × 10^8^ DC_INS_ with 20 ml alginate (w/v, 1.8%; Na-alginate, cat. no. 11061528, Buechi Labortechnik), 200 ml poly(l-lysine) 2000 (w/v, 0.05%; cat. no. 25988-63-0, Alamanda Polymers) solution and using an encapsulator (Inotech Encapsulator IE-50R, EncapBiosystems) set to the following parameters: 200-μm nozzle with a vibration frequency of 1,025 Hz, a 20-ml syringe operated at a flow rate of 400 units and 1.12 kV voltage for bead dispersion. Then, 1.5 ml serum-free DMEM containing 7.5 × 10^6^ microencapsulated cells (500 cells per capsule) was subcutaneously implanted through a 3-ml syringe (cat. no. 9400038, Becton Dickinson) equipped with a 0.7 × 30-mm needle (cat. no. 30382903009009, Becton Dickinson).

### Animal experiments

T1D mice were established by fasting 6-week-old wild-type male Swiss mice (C57BL/6J, Janvier Labs) for 8 h per day for four consecutive days while injecting a single dose per day of freshly diluted streptozotocin (cat. no. S0130, Sigma-Aldrich; 80 mg kg^−1^ in 300 μl sodium citrate buffer (pH 4.3)). T1D-associated persistent fasting hyperglycemia was confirmed after 6 d by 8-h fasting glycemia profiling. For GTTs, treated animals were intraperitoneally injected with 1.5 g kg^−1^ glucose and glycemia was recorded at regular intervals. Blood insulin was quantified using Microtainer serum separator tubes (cat. no. 365967, Becton Dickinson). All experiments involving animals were performed in accordance with the Swiss animal welfare legislation, approved by the veterinary office of the Canton Basel-Stadt, Switzerland (license no. 2996/30779) and conducted by S. Xue (LTK4899) and J. Huang (LTK5912) at the Department of Biosystems Science and Engineering of the ETH Zurich in Basel and according to the directives of the European Community Council (2010/63/EU), approved by the French Republic (project no. DR2018-40v5 and APAFIS no. 16753) and carried out by S. Xue, J. Huang and G. Charpin-El Hamnri (no. 69266309) at the University of Lyon, Institut Universitaire de Technologie. All mice were housed in a 12-h light–dark cycle (five mice per cage). The ambient temperature was 21 ± 1 °C with 50 ± 10% humidity.

#### Sticky patch testing on mice

Electrically conductive double-sticky patches (cat. no. 9701-50, 3M Science,) were taped with wires as electrodes, which were attached to the implantation site for performing electrostimulation.

#### Schedule of mouse experiments

The first electrostimulation was performed 4 h after injecting the microencapsulated cells. For regular glucose monitoring over several weeks, the mice were electrostimulated at midnight with DC 4.5 V for 10 s, then fasted for 6–8 h for glycemia measurement or blood sampling. For glucose monitoring over a whole day at different time points, the mice were electrostimulated at 6:00. For the mouse experiment with multiple electrostimulations per day, the first was performed at midnight, followed by further stimulations at 6-h intervals.

#### Animal blood sampling

Blood samples were taken from the tail or saphenous veins using a 200-µl glass micro-hematocrit capillary (Avantor VWR, cat. no. 521-9100), transferred into blood collection tubes (BD Microtainer, cat. no. BDAM365968) and centrifuged at 8,000*g* for 2 min. The supernatant serum was analyzed or frozen at −80 °C within 1 h following blood collection.

#### Sample preparation for mouse tissue analysis

Wild-type 8-week-old male C57BL/6J mice were injected with encapsulated engineered cells. All stimulated and non-stimulated mice containing two electrodes were killed after 30 min or 48 h of electrostimulation with DC 4.5 V for 10 s and stored in 10% formalin solution (cat. no. HT501128-4L, Sigma-Aldrich).

#### Histology and histopathology

Acupuncture needles were removed and the surrounding tissue was explanted and fixed in 10% neutral-buffered formalin (100 ml 40% formalin, 900 ml ddH_2_O, 4 g l^−1^ NaH_2_PO_4_ and 6.5 g l^−1^ Na_2_HPO_4_, pH 7). The tissue samples were trimmed, dehydrated in increasing concentrations of ethanol, cleared with xylene, infiltrated and embedded in paraffin wax, sectioned at 2–4-µm thickness using an EXAKT 300 CP system (EXAKT Technologies) and stained with hematoxylin and eosin. The tissue sections were analyzed by light microscopy and images were acquired with an Olympus UC30 camera. The tissue around the acupuncture needle footprint was histopathologically evaluated by a pathologist at AnaPath Services according to the ISO 10993-6:2016(E) standard. In addition, collagen denaturation was scored to exclude any potential thermoelectrical impact on the tissues following electrostimulation.

### Reporting summary

Further information on research design is available in the Nature Portfolio Reporting Summary linked to this article.

## Supplementary information

Supplementary Information Supplementary Figs. 1–10, Supplementary Videos 1–3, Supplementary Tables 1–8, Supplementary List and Supplementary References.

Reporting Summary

Supplementary List Supplementary List. Excel file containing four tabs. List S1: gene expression profile for mRNA-seq data (gene analysis information for Extended Data Fig. 4a–f); List S2: overall heat map list, top 500 (gene analysis information for Extended Data Fig. 4e); List S3: antioxidant-related genes in humans (gene analysis information for Extended Data Fig. 4a–f); and List S4: clustering list of expression levels among antioxidant-related genes (gene analysis information for Extended Data Fig. 4f).

Supplementary Video 1 Applying electrostimulation to DART-engineered cells in the incubator. An electrical power source was used to apply DC 5 volts for 10 s. Electrostimulation was performed in a humidified incubator at 37 °C and 5% CO_2_. HEK293 cells were seeded in a 24-well plate and transfected with pJH1003, pJH1004 and pJH1005. To increase the contrast, a white paper was placed under the plate. The wells in the first, third and fifth columns are the control groups without electrostimulation and the wells in the second, fourth and sixth columns are the electrostimulated groups (DC 5 volts for 10 s). Cells were kept in the incubator during the recovery process.

Supplementary Video 2 Fluorescence intensity recording of non-stimulated DART-engineered HEK293 cells. HEK293 cells were transfected with pJH1003, pJH1004 and pJH1214 (P_DART_-slGFP-pA). The video was recorded on a Zeiss LSM 980 Airyscan microscopic system after culturing the cells for 72 h without any stimulation.

Supplementary Video 3 Fluorescence intensity recording of electrostimulated DART-engineered HEK293 cells. HEK293 cells were transfected with pJH1003, pJH1004 and pJH1214 (P_DART_-slGFP-pA). The video was recorded on a Zeiss LSM 980 Airyscan microscopic system after culturing the cells for 72 h following stimulation with DC 5 volts for 20 s.

## Data Availability

The authors declare that all the data supporting the findings of this study are available within the paper and its supplementary materials. All original plasmids listed in Supplementary Table 1 are available upon request. The sequences pJH1003 (GenBank accession no. ON256650), pJH1004 (GenBank accession no. ON256651), pJH1005 (GenBank accession no. ON256652), pJH1054 (GenBank accession no. ON256653), pJH1101 (GenBank accession no. ON256654) and pJH1169 (GenBank accession no. ON256655) are available on GenBank. Source data are provided with this paper.

## Source data

Source Data Fig. 1 Statistical Source Data.
Source Data Fig. 2 Statistical Source Data.
Source Data Fig. 3 Statistical Source Data.
Source Data Fig. 4 Statistical Source Data.
Source Data Fig. 5 Statistical Source Data.
Source Data Fig. 6 Statistical Source Data.
Source Data Extended Data Fig. 1 Statistical Source Data.
Source Data Extended Data Fig. 2 Statistical Source Data.
Source Data Extended Data Fig. 3 Statistical Source Data.
Source Data Extended Data Fig. 5 Statistical Source Data.
Source Data Extended Data Fig. 6 Statistical Source Data.
Source Data Extended Data Fig. 6 Unprocessed western blot gels.
Source Data Extended Data Fig. 7 Statistical Source Data.
Source Data Extended Data Fig. 8 Statistical Source Data.
Source Data Extended Data Fig. 9 Statistical Source Data.
Source Data Extended Data Fig. 10 Statistical Source Data.

## Code availability

The code used in this paper is publicly available on GitHub (https://github.com/Jinbo2022).
