# Supplementary material for: An electrogenetic interface to program mammalian gene expression by direct current
Source: Nat Metab. 2023 Jul 31;5(8):1395–407. doi: 10.1038/s42255-023-00850-7 (PMC10447240; doi:10.1038/s42255-023-00850-7)
Supplement: Supplementary file 1 — Supplementary Figs. 1–10, Supplementary Videos 1–3, Supplementary Tables 1–8, Supplementary List and Supplementary References. [file 42255_2023_850_MOESM1_ESM.pdf]

nature metabolism

Article https://doi.org/10.1038/s42255-023-00850-7

An electrogenetic interface to program mammalian gene expression by direct current

In the format provided by the authors and unedited

Supplementary Figures

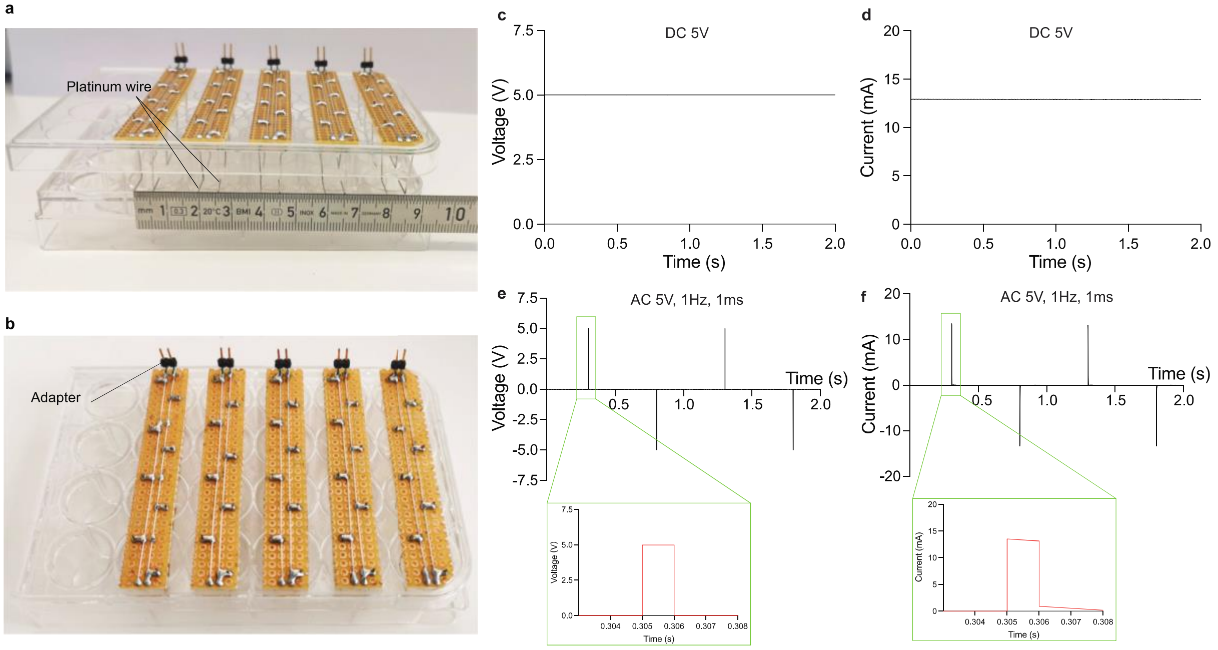

Supplementary Figure 1. Design of cell culture plate with customized lid and measurement of the applied voltage and current. a, Front view of the plate showing L-shaped 0.5 mm platinum electrodes attached to the lid at a separation distance of 6 mm. **b**, Bird's-eye view of the customized lid of the 24-well plate. The adapter was designed to connect to a power source. **c-f,** measurement of the applied voltage and current generated in the culture medium for DC and AC programs. **c**,**d**, Voltage generated by the DC power supply (**c**) and the corresponding current (**d**) produced in the culture medium inside the stimulation plates (**a,b**) during 2 s when applying 5 volts. **e**, Square voltage generated by the AC pulse generator (HP3245A) during 2 s when programmed to deliver 1 ms pulses of 5 volts at a frequency of 1 Hz. The expanded plot shows the first square wave. **f**, The current produced in the culture medium when applying square AC (5 V, 1 Hz, 1 ms). The expanded plot shows the first square waveform of current. Voltages and currents in the culture medium were measured using a potentiostat (CHI760E).

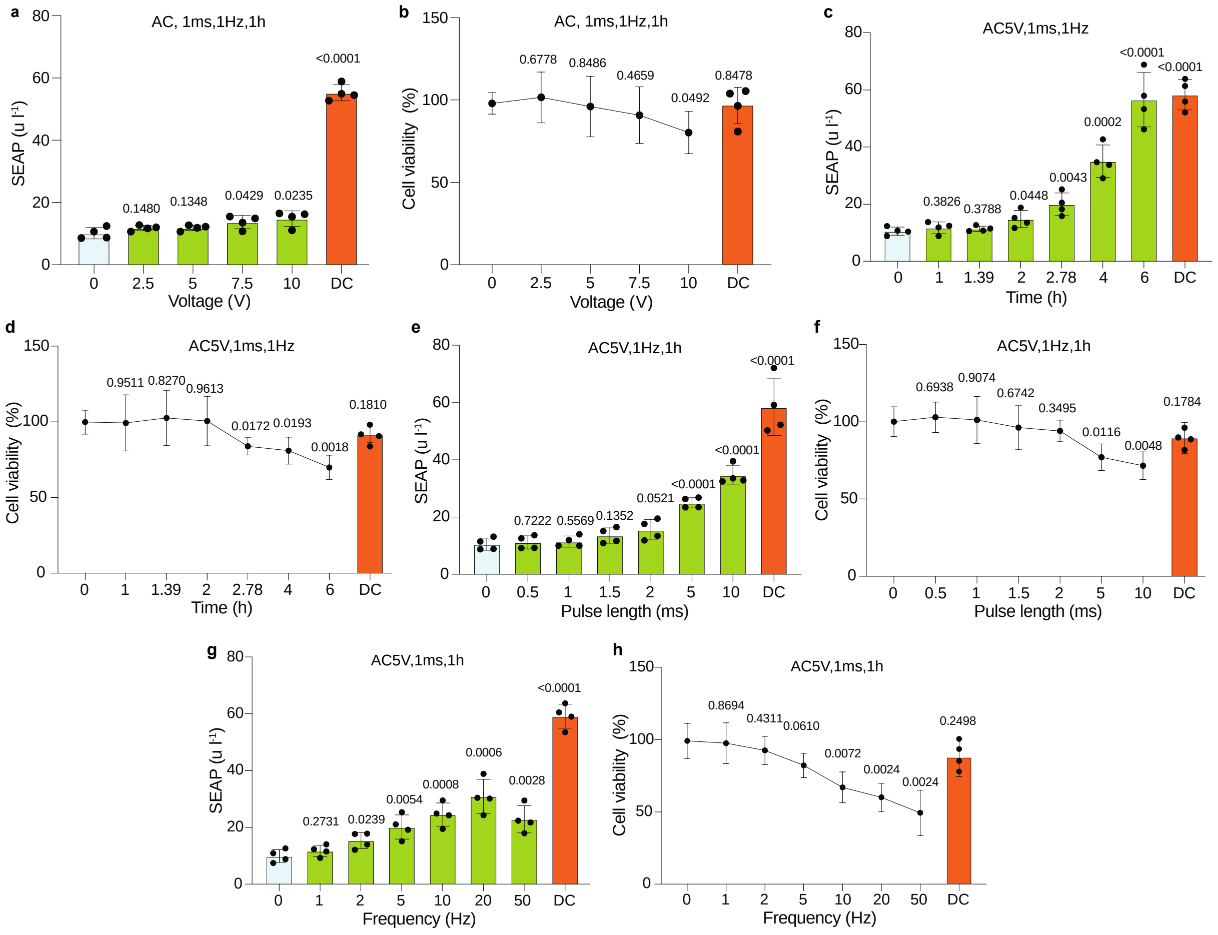

Supplementary Figure 2. Testing of different alternating current (AC) pulse programs. **a**,**b**, SEAP production and viability of DART-engineered cells measured 24 hours after stimulation. **a**,**b**, Voltage dependence. Pulses of 1 ms were applied at the indicated voltage for 1 hour at 1 Hz. **c**,**d**, Duration of electrical stimulation. Pulses of 1 ms at 1 Hz and 5 volts were applied during the indicated time periods. The power produced from DC 5 V for 20 s equals that of AC 5 V with 1 Hz frequency and 1 ms pulse length applied during 2.78 h, according to Ohm's Law, with the square waveform shown in supplementary Figure 2. **e**,**f**, Effect of pulse length. Electrical stimulation was performed at 5 volts for 1 hour at 1 Hz, with the indicated pulse length. **g**,**h**, Effect of frequency. Electrical stimulation was performed at 5 volts for 1 hour with 1 ms pulse length at the indicated frequencies. The samples marked as DC in all panels represent the positive control (stimulation at 5 volts DC for 20 s). Bars in panels (**a**), (**c**), (**e**) and (**g**) represent mean ± SD; n = 4; black circles show the individual measurements. The orange bar represents the positive control (stimulation at 5 volts DC for 20 s). Data points in panels (**b**), (**d**), (**f**), (**h**) represent mean ± SD; n = 4. *P* values were calculated between stimulated and non-stimulated groups.

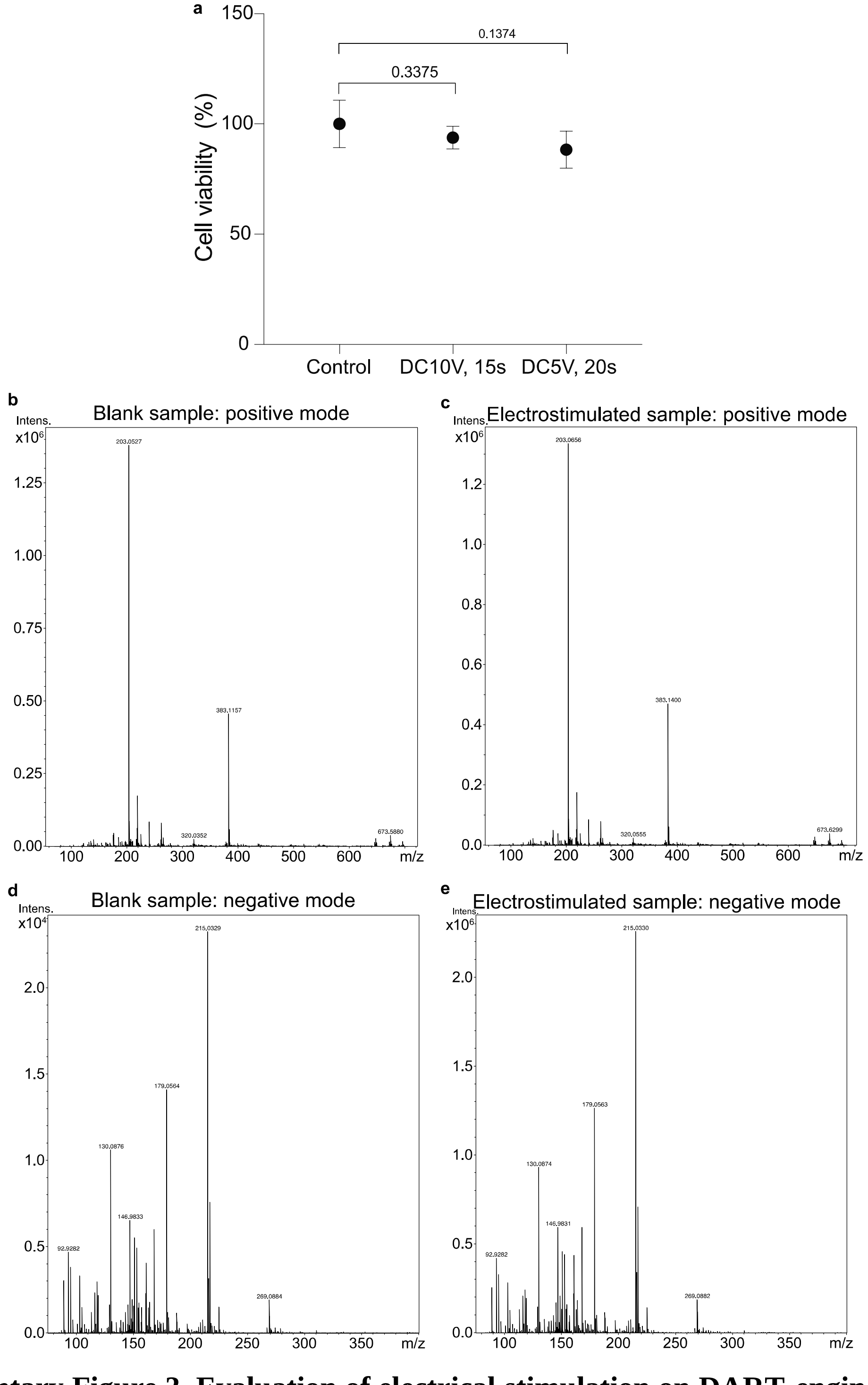

Supplementary Figure 3. Evaluation of electrical stimulation on DART-engineered cells. **a,** Impact of electrical stimulation on the viability of DART-engineered cells. The cell viability was analyzed after stimulation with direct current (DC) at 10 volts for 15 s (DC10V, 15s) and 5 volts for 20 s (DC5V, 20s), and compared with that of non-stimulated cells (Control) taken as 100%. **b-e,** Electrospray ionization mass spectrometry (ESI-MS) analysis of the medium. **b**,**c**, Analysis of non-electrostimulated medium in positive (**b**) and (**c**) negative ion polarity modes. **d**,**e**, Positive (**d**) and negative (**e**) ion polarity analyses of medium treated at 10 volts for

30 seconds. Data points represent mean ± SD; n = 4. *P* values were calculated between stimulated and non-stimulated groups.

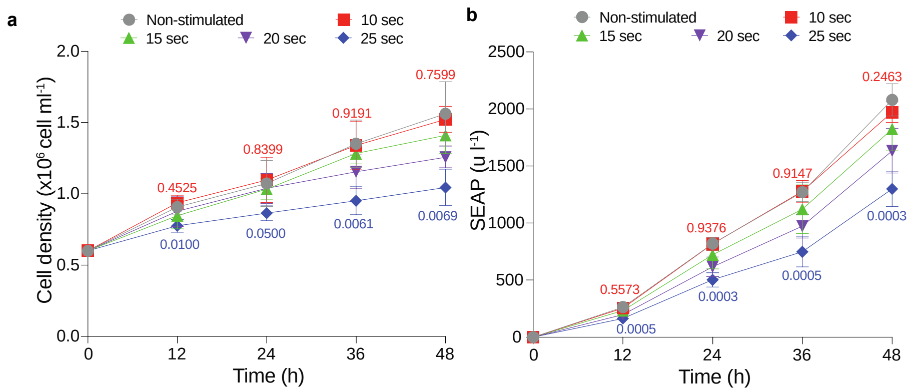

Supplementary Figure 4. Impact of electrostimulation of DART-engineered HEK-293 cells on cell growth and recombinant protein production. a,**b**, Time courses of cell density (**a**) and SEAP secretion (**b**) after electrical stimulation at 5 volts for 10, 15, 20 and 25 s. HEK-293 cells with constitutive expression of SEAP (transfected with pJH3, P_{hCMV}-SEAP-pA) were electrically stimulated and analyzed every 12 h during 48 h of culture. Data points represent mean ± SD, n = 4. The statistical designations with different colors refer to the corresponding data points with the same color.

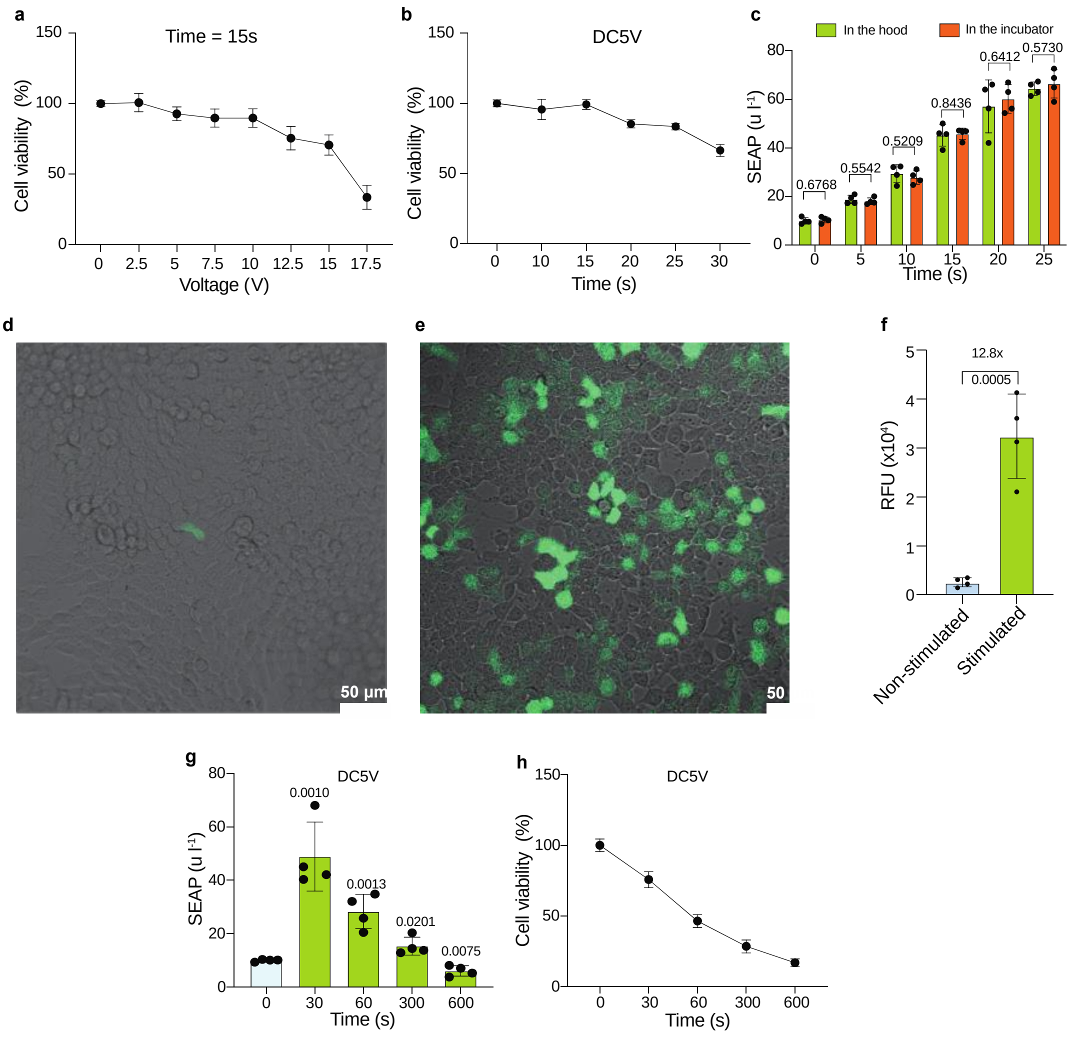

Supplementary Figure 5. Evaluation of electrostimulation on DART-engineered HEK-293 cells. a, Impact of electrostimulation on viability. **a**,**b**, Cells were electrostimulated for 15 s at the indicated DC voltages (**a**), or at 5 volts DC for the indicated time periods (**b**), and viability was measured using a resazurin assay. **c,** Performance comparison of DART-engineered cells electrostimulated outside or inside of a cell-culture incubator. Supernatant SEAP levels were measured 24 hours after electrostimulation at DC 5 volts during the indicated time periods, in the laminar flow hood (green) or inside the incubator (orange). Cell cultures stimulated outside the incubator were returned to the incubator (humidified at 37 °C and 5% CO_2) immediately after electrostimulation. **d-f,** Fluorescence intensity analysis of DART-engineered HEK-293 cells encoding a short-lived eGFP (slGFP) downstream of the DART promoter. **d**,**e**, HEK-293 cells were transfected with pJH1003, pJH1004 and pJH1214 (P_{DART}-slGFP-pA). Microcopy images of non-stimulated (**d**) or stimulated cultures (**e**), 72 hours after applying DC 5 volts for 20 s. **f**, eGFP fluorescence intensity analysis from non-stimulated and

stimulated cultures, 72 hours after applying DC 5 volts for 20 s. RFU, relative fluorescence units. The images were taken by a Zeiss LSM 980 Airyscan microscopic system. **g,** Effect of exposure time of DART-engineered HEK-293 cells to stimulation at 5 volts DC (DC5V) on SEAP production (**g**) and cell viability (**h**). Data points represent mean ± SD; n = 4.

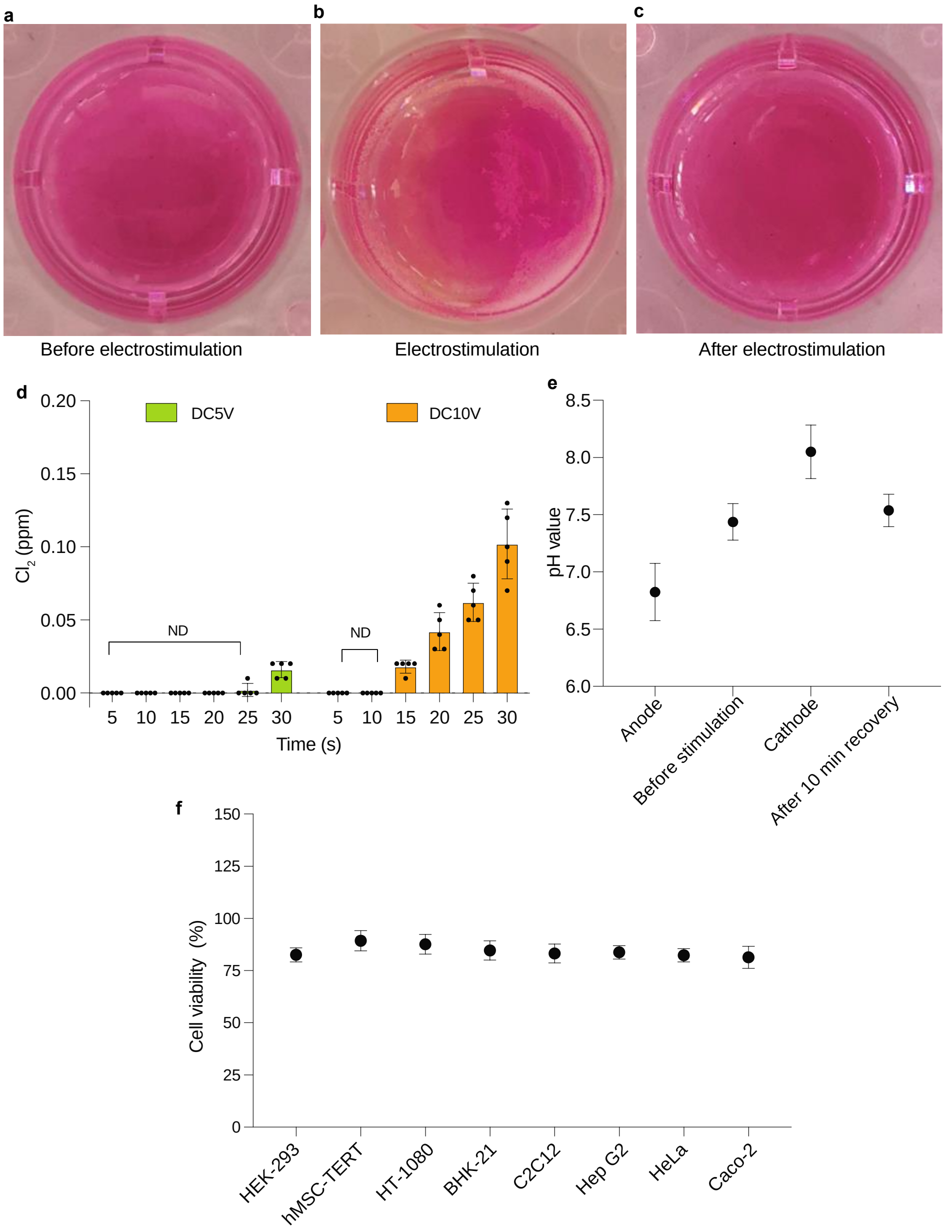

Supplementary Figure 6. Impact of electrostimulation on culture medium and viability of mammalian cells. a,**b**, Images of the culture medium in one well of a 24-well plate before (**a**) and immediately after (**b**) electrostimulation at 5 volts DC for 30 s. **c**, Electrostimulated medium after about 10 min. **d**, Analysis of Cl_2 produced during electrostimulation at 5- or 10-volts DC for the indicated time periods, in wells of a 24-well plate containing 750 µl of culture medium. **e**, pH of the medium around the anode and cathode was measured before and after electrostimulation, and after 10 min of recovery following electrostimulation. **f,** viability of various DART-transfected mammalian cells electrostimulated at 5 volts DC for 20 s. Viability was measured using resazurin assay. Data points represent mean ± SD;n = 5 in (**d**), n = 8 in (**e**) and n = 4 in (**f**). ND means not detectable.

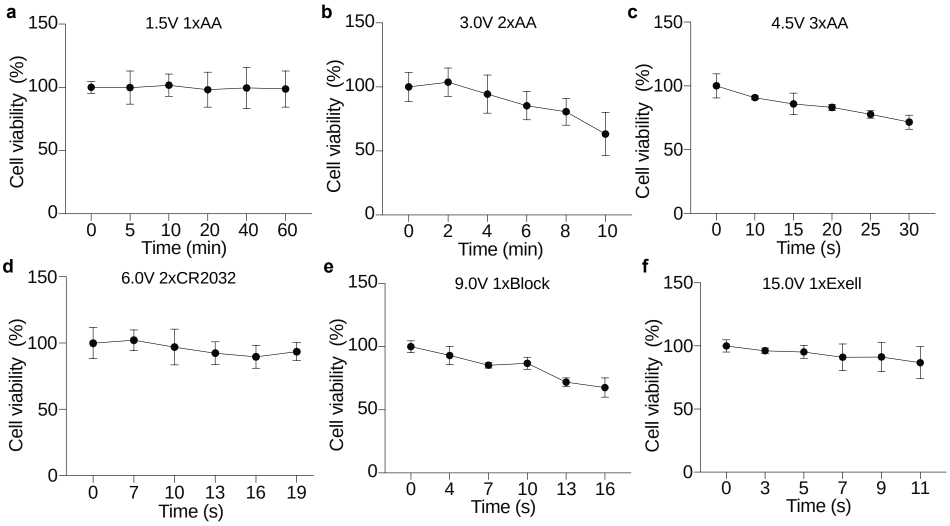

Supplementary Figure 7. Viability of DART-engineered HEK-293 cells stimulated with alkaline batteries or other DC power sources. a-f, Electrostimulation with the indicated batteries. Viability was assessed at 24 h after electrostimulation for the indicated time. Data points represent mean ± SD; n = 4.

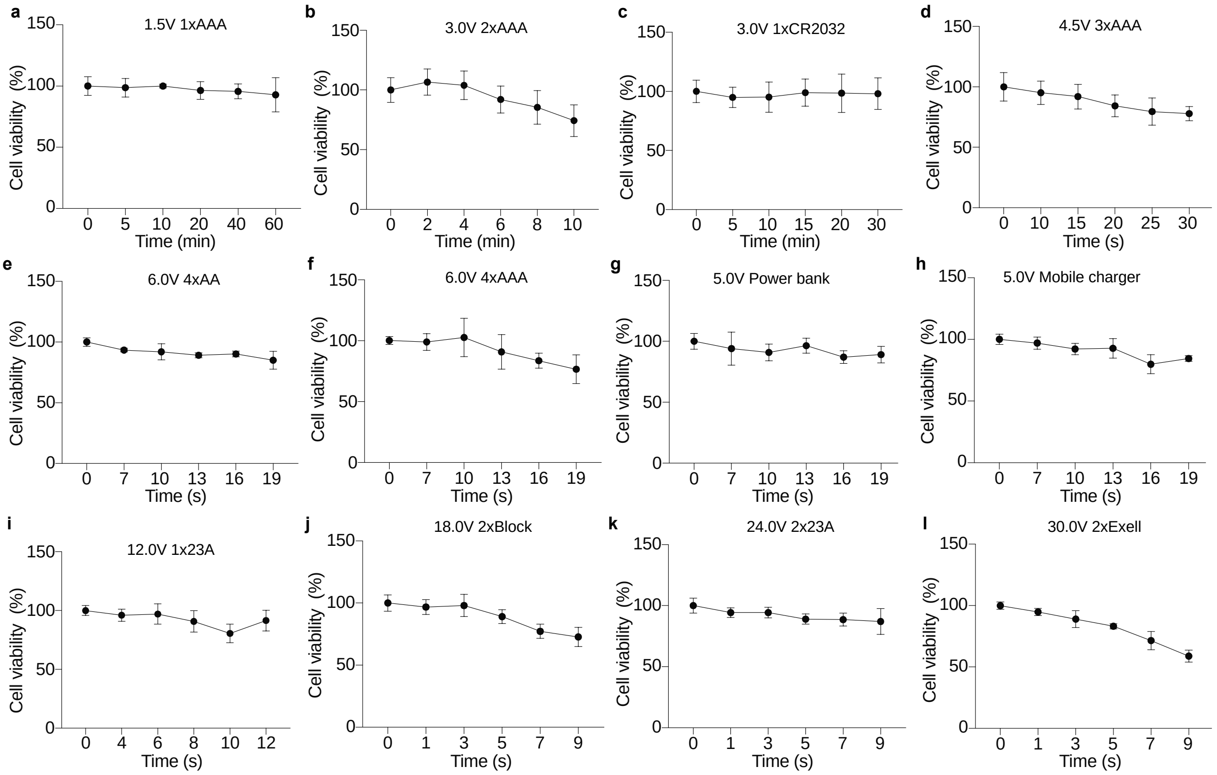

Supplementary Figure 8. Viability of DART-engineered HEK-293 cells stimulated with direct current from alkaline batteries. a-f and **i-l**, Cells were electrostimulated using the indicated batteries. **g**,**h**, Electrostimulation with a power bank (**g**) or a mobile charger (**h**). Viability was assessed at 24 hours after electrostimulation for the indicated time. Data points represent mean ± SD; n = 4.

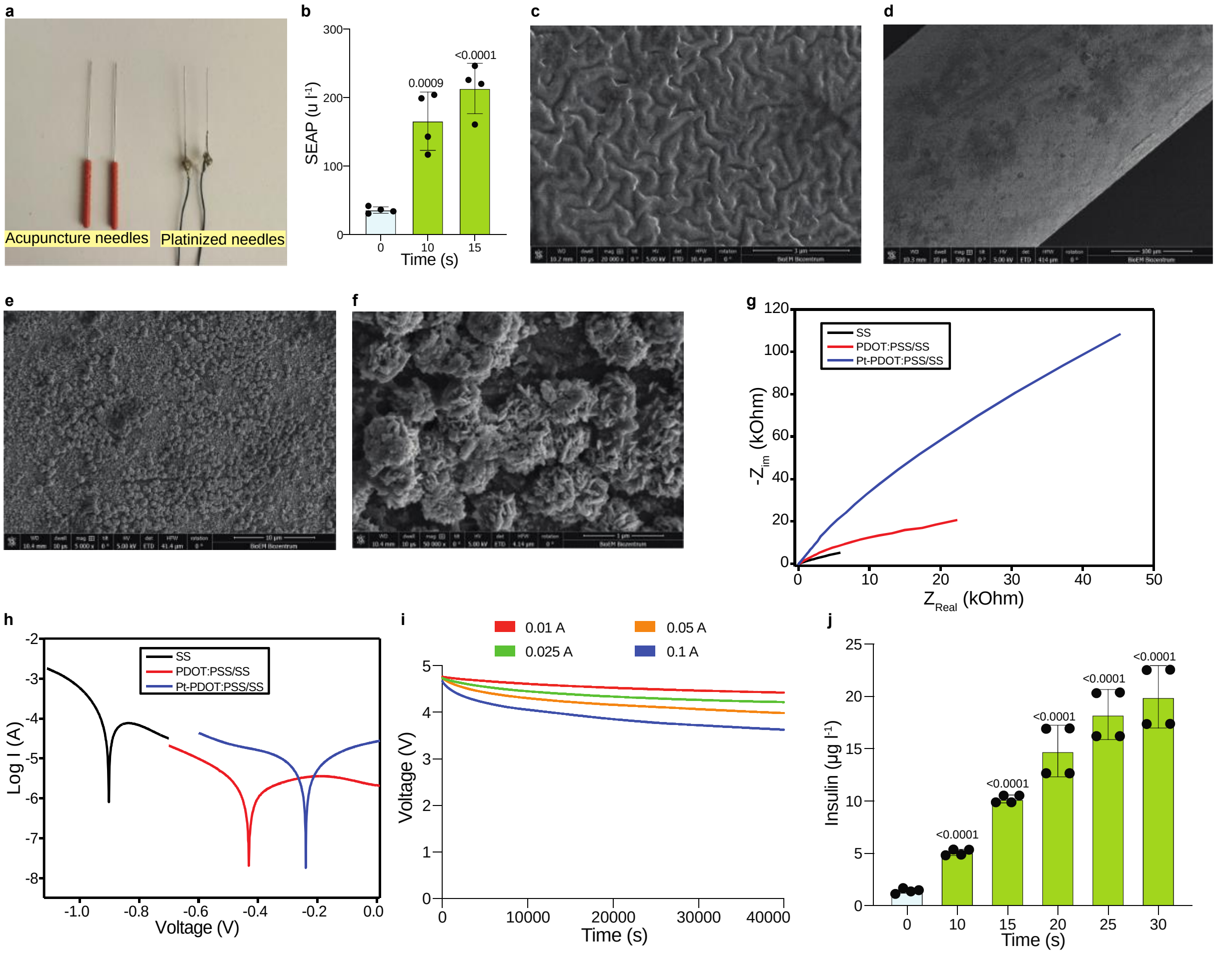

Supplementary Fig. 9. Platinized acupuncture needles can be used to stimulate DC$_{INS}$ cell cultures. a, Pictures of the original acupuncture needles (left) and platinized acupuncture needles (right). **b**, SEAP produced by cells stimulated at 4.5 volts DC for the indicated time with platinized acupuncture needles. **c-h,** Preparation of platinized acupuncture needles. **c**, SEM image of uniformly deposited PDOT:PSS/SS before Pt deposition (20,000× magnification). **d-f**, Scanning electron microscope (SEM) images of Pt-PDOT:PSS/SS electrodes used in experiments (500×, 5000× and 50,000× magnification, respectively), showing Pt nanosphere clusters. Scale bars are shown at the bottom right underneath the images in (**c-f**). **g**, Nyquist plots for deposited samples at different stages of modification. The behavior of the modified electrode was mostly resistive, suggesting that strong polymer bonds and Pt modification on the acupuncture needle surface prevent corrosive agents from penetrate beneath the coating. The coated acupuncture needle (SS) was washed by ultrasonication for 10 min in a solution of ethanol (v/v, 75%). The platinized acupuncture needle was used as the anode and platinum wire (1 mm diameter) as the counter electrode. **h**, Tafel plots for platinized acupuncture needles at different stages of modification. From the measured I$_{corr}$ values, the protection efficiency (PE) was obtained from the following equation: $PE = ((I_{corr} - I_{corr(c)})/I_{corr})$

x 100 (1), in which I_{corr} and $I_{corr(c)}$ are the corrosion current density values in the absence and presence of the coating, respectively. The experiments were repeated independently with similar results. **i,** Battery testing. Discharge curves for AA batteries with different anode currents. Three tandem AA batteries were connected to a battery tester and discharged with currents of 0.01, 0.025, 0.05 and 0.1 A. The output voltage was decreased by about 7.5%, 11.3%, 16.6% and 23.2%, respectively, after continuous discharge for 10 h. The voltage was recorded every 10 s for 10 h. **j,** Insulin production by alginate-encapsulated DC_{INS} cells. The cells were stimulated at 4.5 volts DC for the indicated time periods. Data points represent mean ± SD; n = 4.

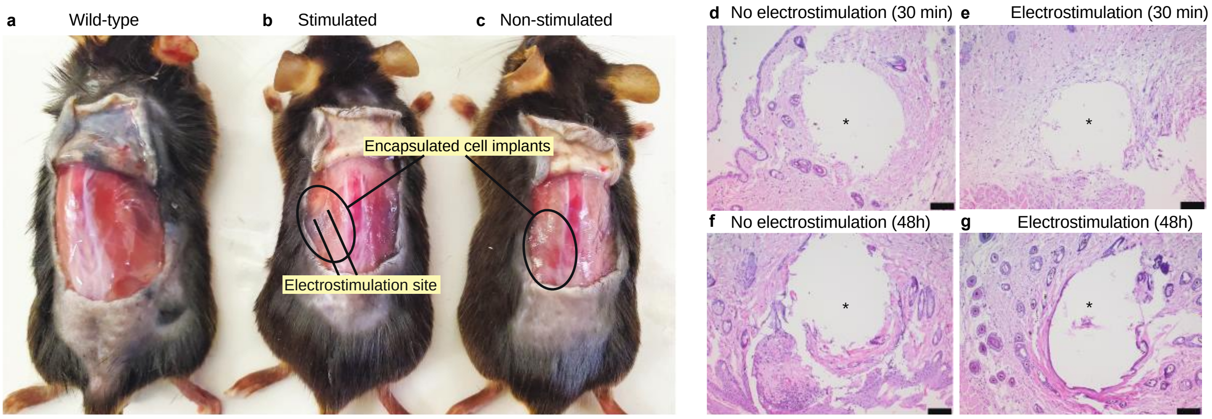

Supplementary Figure 10. Macroscopic and histological analysis of tissue around the implantation site. a-c, Representative images of wild-type **(a)**, stimulated (**b**) and non-stimulated (**c**) mice. The mice were subcutaneously injected with encapsulated alginate beads containing DART-engineered cells on the dorsal site (indicated by the black circles). The mouse in (**b**) was electrostimulated by platinized acupuncture needles (indicated by black lines) at the implantation site using DC 4.5 volts for 10 s per day, for four consecutive weeks. **d-g,** Histological analysis of tissue sections around the dermal acupuncture needle implantation site. Comparative analysis of non-stimulated (**d**, **f**) and electrostimulated (**e**, **g**; 10 s, DC 4.5 volts) dermal samples 30 min (**d**, **e**) and 48 h (**f**, **g**) after the treatment intervention reveals no significant differences in host reactions, with no detectable immune cell infiltration, fatty infiltration, fibrosis, edema, hemorrhage, neovascularization or necrosis around the platinized acupuncture needle footprint (indicated by an asterisk). Hematoxylin and eosin staining,

original objective lens 10x, black scale bars = 100 µm. In **d**-**g**, n=4, the experiment was not repeated.

Supplementary movies

Supplementary movie 1. Applying electrostimulation to DART-engineered cells in the incubator. An electrical power source was used to apply DC 5 volts for 10 s. The electrostimulation was performed in a humidified incubator at 37 °C and 5% CO_2. The HEK-293 cells were seeded in a 24-well plate and transfected with pJH1003, pJH1004 and pJH1005. To increase the contrast, a white paper was placed under the plate. The wells in the first, third and fifth columns are the control groups without electrostimulation, and the wells in the second, fourth and sixth columns are the electrostimulated groups (DC 5 volts for 10 s). Cells were kept in the incubator during the recovery process.

Supplementary movie 2. Fluorescence intensity recording of non-stimulated DART-engineered HEK-293 cells. HEK-293 cells were transfected with pJH1003, pJH1004 and pJH1214 (P_{DART}-slGFP-pA). The video was recorded on a Zeiss LSM 980 Airyscan microscopic system after culturing the cells for 72 hours without any stimulation.

Supplementary movie 3. Fluorescence intensity recording of electrostimulated DART-engineered HEK-293 cells. HEK-293 cells were transfected with pJH1003, pJH1004 and pJH1214 (P_{DART}-slGFP-pA). The video was recorded on a Zeiss LSM 980 Airyscan microscopic system after culturing the cells for 72 hours following stimulation with DC 5 volts for 20 s.

Supplementary Table 1. Plasmids used and designed in this study

Plasmid	Description	Reference
BB6-BlastR	SB100X-specific transposon containing a constitutive BlastR and iRFP expression unit (ITR-MCS:P_{hCMV}-BlastR-P2A-iRFP-pA-ITR).	Unpublished
BB6-PuroR	SB100X-specific transposon containing a constitutive ECFP and PuroR expression unit (ITR-MCS:P_{RPBSA}-ECFP-P2A-PuroR-pA-ITR).	Unpublished
BB6-ZeoR	SB100X-specific transposon containing a constitutive ZeoR and mRuby expression unit (ITR-MCS-pA: P_{hCMV}-ZeoR-P2A-mRuby-pA-ITR).	Unpublished
H107	Constitutive mammalian expression vector. (P_{hCMV}-eGFP-3FLAG:P_{mPGK}-ZeoR-pA).	ObiO, Shanghai
Mkp37	Constitutive mammalian TetR-Elk1 fusion protein expression vector (P_{hCMV}-TetR-Elk1-pA).	Keeley et al.[1]
pcDNA3.1(+)	Constitutive mammalian expression vector containing a NeoR resistance gene (P_{hCMV}-MCS-pA).	Thermo Fisher Scientific, CA
pCMV-T7-SB100	Constitutive SB100X expression vector (P_{hCMV}-SB100X-pA) (Addgene no. 34879).	Mates et al.[2,3]
pCK53	CRE-driven SEAP expression vector (P_{CRE}-SEAP-pA).	Kemmer et al.[4]
pdCas9-VPR	Constitutive dCas9-VPR expression vector (P_{hCMV}- dCas9-VPR-pA) (Addgene no. 63798).	Chavez et al.[5]
pKEAP1	Constitutive KEAP1 expression vector (P_{hCMV}-KEAP1-3xFlag-pA) (Addgene no. 28023).	Fan et al.[6]

pLeo1403	Mammalian reporter plasmid for TetR-Elk1-induced NanoLuc expression (O_{TetR}-$P_{hCMVmin}$-NanoLuc-pA).	Maysam et al.[7]
pMF111	Mammalian reporter plasmid for TetR-Elk1-induced SEAP expression (P_{TRE}-SEAP-pA).	Fussenegger et al.[8]
pMX256	SB100X-specific transposon containing a P_{NFAT5}-driven SEAP and mouse insulin expression unit and a constitutive eGFP and ZeoR expression unit (ITR-P_{NFAT5}-SEAP-P2A-mINS-pA:P_{RPBSA}-eGFP-P2A-ZeoR-pA-ITR).	Xie et al.[9]
pNRF2	Constitutive NRF2 expression vector (P_{hCMV}-Flag-NRF2-pA) (Addgene no. 36971).	Camp et al.[10]
pXS101	SB100X-specific transposon containing a constitutive mammalian promotor-driven CREB1-TetR stable expression vector in mammalian cells (ITR-P_{hCMV}-CREB1-TetR-2A-mCherry-2A-PuroR-pA-ITR).	Xue et al.[11]
pJH3	Constitutive mammalian SEAP expression vector (P_{hCMV}-SEAP-pA). The fragment was PCR-amplified from pCK53 with OJH3-GF (5'-CGAAGCGGAATTCACCATGACT AGTCTGCTGCTGCTGCTG-3') and OJH3-GR (5'-TTTCTAGACACCGGTGGATCCGCTAGCGGT CTGCTCGAATCTGCC-3'), and cloned into pcDNA3.1 (digested by *Spe*I/*Bam*HI) by Gibson assembly[12].	This work
pJH42	Constitutive SB100X expression vector (P_{hCMV}-SB100X-pA). The fragment was PCR-amplified from pCMV-T7-SB100 (Addgene no. 34879) with OJH42-GF (5'-AGCGGAATTCACCATGACTAGTAAATCAAAAGAA ATCAGCCAAGACC-3') and OJH42-GF (5'-TTCTAGACACCGGTGGATCCCTAGTATTTGGTAGC ATTGCCTTTAAATTG-3'), and cloned into pJH3 (digested by *Spe*I/*Bam*HI) by Gibson assembly.	This work

pJH1003	Constitutive NRF2 expression vector (P_{hCMV}-NRF2-pA).<br>The fragment was PCR-amplified from FLAG-NRF2 (Addgene no. 36971) with OJH1003-GF (5'- GCG GAATTCACCATGACTAGTATGATGGACTTGGAGCT GCC-3') and OJH1003-GR (5'- TTTCTAGA CACCGGTGGATCCCTAGTTTTTCTTAACATCTGGC TTCTTACTTTTG-3'), and cloned into pJH3 (digested by *Spe*I/*Bam*HI) by Gibson assembly.	This work
pJH1004	Constitutive KEAP1 expression vector (P_{hCMV}-KEAP1-pA).<br>The fragment was PCR-amplified from FLAG-KEAP1 (Addgene no. 28023) with OJH1004-GF (5'- GC GGAATTCACCATGACTAGTATGCAGCCAGATCCC AGGC-3') and OJH1004-GR (5'- TCTAGAC ACCGGTGGATCCCTAACAGGTACAGTTCTGCTGGT CAATC-3'), and cloned into pJH3 (digested by *Spe*I/*Bam*HI) by Gibson assembly.	This work
pJH1005	ARE-driven SEAP expression vector (P_{DART}-SEAP-pA).<br>The fragment was PCR-amplified from pJH3 with ARE-containing primer OJH1005-GF (5'-CGGATC GGGAGATCTCCACGCGTGTCAGTCACAGTGACTC AGCAGATATCTCGAGGGTAGGCGTGTACG-3') and OJH1005-GR (5'-TTTCTAGACACCGGTGGATCCCTAGGTCTGCTCGA ATC TGCCG-3'), and cloned into pJH3 (digested by *Mlu*I/*Bam*HI) by Gibson assembly.	This work
pJH1006	Two tandem ARE-driven SEAP expression vector (P_{DART2}-SEAP-pA).<br>The fragment was PCR-amplified from pJH1005 with OJH1006-GF (5'-TCGGGAGATCTCCACGCGT	This work

	GTCAGTCACAGTGACTCAGCAGATATGAGTATAA GTCAGTCACAGTGACTCAGCAGATAT-3') and OJH1005-GR (5'-TTTCTAGACACCGGTGGATCCCTAGGTCTGCTCGA ATCTGCCG-3'), and cloned into pJH3 (digested by *Mlu*I/*Bam*HI) by Gibson assembly.	
pJH1009	Three tandem ARE-driven SEAP expression vector (P_{DART3}-SEAP-pA).<br>The fragment was amplified by two rounds of PCR. The first round of PCR was amplified from pJH1005 with OJH1009-GF1 (5'-CAGCAGATATCACAATAGGTCAGTCACAGTGACT CAGCAGATATCCA CGCGTGTCAGTCACAGTGACTCAGCAGATATC-3') and OJH3-SEAP-GR (5'-AAGCTTACACCG GTGGATCCGCTAGCTGGTCTGCTCGAATCTGCC-3'), and the second round of PCR was amplified from the product that was recovered in the first round with OJH1009-GF2 (5'-CGGATCGGGAGATCT CCACGCGTGTCAGTCACAGTGACTCAGCAGATATC ACAATAGGTCAGTCAC-3') and OJH1005-GR (5'-TTTCTAGACACCGGTGGATCCCTAGGTCTGCTCGA ATCTGCCG-3'), then the fragment from the second round of PCR was cloned into pJH3 (digested by *Mlu*I/*Bam*HI) by Gibson assembly.	This work
pJH1010	Four tandem ARE-driven SEAP expression vector (P_{DART4}-SEAP-pA).<br>The fragment was PCR-amplified from pJH1009 with OJH1010-GF (5'-TCGACGGATCGGGAGATC TCCACGCGTCAGTCAGTGTGTGACTCAGCACTCTT ATCGGGAGATCTCCACGCGTGTCAG-3') and OJH1005-GR (5'-	This work

	TTTCTAGACACCGGTGGATCCCTAGGTCTGCTCGA ATCTGCCG-3'), and cloned into pSEAP-control (digested by *Mlu*I/*Bam*HI) by Gibson assembly.	
pJH1053	SB100X-specific transposon containing a constitutive NRF2 and ZeoR expression unit (ITR-P_{hCMV}-NRF2-P2A-ZeoR-pA-ITR). The CMV-NRF2 fragment was PCR-amplified from pJH1003 with OJH1053-1054-GF (5'-GTTTCGGT AAGGGGTCCGCTAACGCGTGGTACCCTCGAGTTG-3') and OJH1053-Nrf2-GR (5'-CCTCGACG TCACCAGCCTGCTTCAGCAGGCTGAAGTTAGTAGC GCCGGAGCCGTTTTTCTTAACATCTGGCTTCTTAC-3'), and the P2A-ZeoR fragment was PCR-amplified from H107 with OJH1053-Zeo-GF (5'-CTACTAACTTCAGCCTGCTGAAGCAGGCTGGTGAC GTCGAGGAGAATCCTGGTCCCATGGCCAAGTTGA CCAGTGC-3') and OJH1053-GR (5'-CAAGCTTCACGACAGGCCTTCGAATCAGT CCTGCTCCTCGGCC-3'), then the two fragments were cloned into pXS101 (digested by *Mlu*I/*Bst*BI) by Gibson assembly.	This work
pJH1054	SB100X-specific transposon containing a constitutive KEAP1 and BlastR expression unit (ITR-P_{hCMV}-KEAP1-P2A-BlastR-pA-ITR). The CMV-KEAP1 fragment was PCR-amplified from pJH1004 with OJH1053-1054-GF (5'-GTTTCGG TAAGGGGTCCGCTAACGCGTGGTACCCTCGAGTTG -3') and OJH1054-Keap1-GR (5'-CTCGAC GTCACCAGCCTGCTTCAGCAGGCTGAAGTTAGTAG CGCCGGAGCCACAGGTACAGTTCTGCTGGTC-3'), and the P2A-BlastR fragment was PCR-amplified from BB6-Zeocin with OJH1054-Blast-GF (5'-	This work

	ACTAACTTCAGCCTGCTGAAGCAGGCTGGTGACGT CGAGGAGAATCCTGGTCCCAT GGCCAAGCCTTTGTCTC-3’) and OJH1054-GR (5’-GCTTCACGACAGGCCTTCGAACTAGCCCT CCCACACATAACC-3’), then the two fragments were cloned into pXS101 (digested by *Mlu*I/*Bst*BI) by Gibson assembly.	
pJH1089	ARE-driven NanoLuc expression vector (P_{DART}-NanoLuc-pA).<br><br>The fragment was PCR-amplified from pLeo1403 with OJH1089-GF (5’-AAGCGGAATTCACCATGA CTAGTGAGACAGACACACTCCTGC-3’) and OJH1089-GR (5’-AGCTTTCTAGACACCGGTGGAT CCCTACGCCAGAATGCGTTCGCAC-3’), and cloned into pJH1005 (digested by *Spe*I/*Bam*HI) by Gibson assembly.	This work
pJH1090	ARE-driven NanoLuc and mouse insulin expression vector (P_{DART}-NanoLuc-P2A-mINS-pA).<br><br>The NanoLuc fragment was PCR-amplified from pLeo1403 with OJH1089-GF (5’-AAGCGGAATTCA CCATGACTAGTGAGACAGACACACTCCTGC-3’) and OJH1090-NanoLuc-GR (5’-CAGCAGGGA AAAGTTGGTTGCTCCTGTCTGCGCCAGAATGCGTT C-3’), and the P2A-mINS fragment was PCR-amplified from pMX256 with OJH1090-P2A-GF (5’-GGAGCAACCAACTTTTCCCTGCTGAAGCAG GCA-3’) and OJH1090-mINS-GR (5’-AAGCTTTCTAGACACCGGTGGATCCTCAGTTGCAG TAGT TCTCCAGTTGG-3’), then the two fragments were cloned into pJH1005 (digested by *Spe*I/*Bam*HI) by Gibson assembly.	This work

pJH1096	SB100X-specific transposon ARE-driven NanoLuc and mouse insulin expression unit, and a constitutive ZeoR expression unit<br>(ITR- ITR-P_{DART}-NanoLuc-P2A-mINS: mPGK-ZeoR-pA-ITR)<br>The target fragment was PCR-amplified from pJH1090 with OJH1096-IGF (5'-CGGATCGGGAGATC TCCACGCGTGTCAGTCACAGTGACTCAGCA-3') and OJH1096-IGR (5'-CCTACCCGGTAGAAT TATCTAGATTAGTTGCAGTAGTTCTCCAGTTGGTA G-3'), and the backbone fragment was PCR-amplified from H107 with OJH1096-VGF (5'-CTACCAACTGGAGAACTACTGCAACTAATCTAGA TAATTCTACCGGGTAGGGGAG-3') and OJH1096-VGR (5'-GCGTGGAGATCTCCCGATCCGTA GCGGACCCCTTACCGAAAC-3'), then the two fragments were combined by Gibson assembly.	This work
pJH1101	SB100X-specific transposon containing a constitutive NRF2 expression unit and a constitutive ECFP and PuroR expression unit<br>(ITR-P_{hCMV}-NRF2-pA: P_{RPBSA}-ECFP-P2A-PuroR-pA-ITR).<br>The fragment was PCR-amplified from pJH1003 with OJHBB3-BB6-GF (5'-CCGCTATCTAGTCTTA AGAGATCTACGCGTGGTACCCTCGAGTTG-3') and OJHBB3-BB6-GR (5'-GGCCTGCAGGCCG GCCTCAAAGCTTTCTAGACACCGGTGGATC-3'), and cloned into BB6-PuroR (digested by *Mlu*I/ *Hin*dIII) by Gibson assembly.	This work

pJH1102	SB100X-specific transposon containing a constitutive KEAP1 expression unit and a constitutive BlastR and iRFP expression unit<br>(ITR-P_{hCMV}-KEAP1-pA:P_{hCMV}-BlastR-P2A-iRFP-pA-ITR).<br>The fragment was PCR-amplified from pJH1004 with OJHBB3-BB6-GF (5'-CCGCTATCTAGTCTTA AGAGATCTACGCGTGGTACCCTCGAGTTG-3') and OJHBB3-BB6-GR (5'-GGCCTGCAGGCCG GCCTCAAAGCTTTCTAGACACCGGTGGATC-3'), and cloned into BB6-BlastR (digested by *Mlu*I/ *Hin*dIII) by Gibson assembly.	This work
pJH1157	Four tandem ARE-driven SEAP and mouse insulin expression vector (P_{DART4}-SEAP-P2A-mINS-pA)<br>The fragment was PCR-amplified from pMX256 with OJH1157-GF (5'-CAAGCTGTTCGAAGCGGA ATTCGCCACCATGCTGCTGCTG-3') and OJH1157-GR (5'-TTTCTAGACACCGGTGGATCCCTA GTTGCAGTAGTTCTCCAGTTGGTA-3'), and cloned into pJH1010 (digested by *Eco*RI/*Bam*HI) by Gibson assembly.	This work
pJH1159	SB100X-specific transposon containing a four tandem ARE-driven SEAP and insulin expression unit and a constitutive ECFP and PuroR expression unit<br>(ITR-P_{DART4}-SEAP-P2A-mINS-pA:P_{RPBSA}-ECFP-P2A-PuroR-pA-ITR).<br>The fragment was PCR-amplified from pJH1010 with OJHBB3-BB6-GF (5'-CCGCTATCTAGTCTTA AGAGATCTACGCGTGGTACCCTCGAGTTG-3') and OJHBB3-BB6-GR (5'-GGCCTGCAGGCCG GCCTCAAAGCTTTCTAGACACCGGTGGATC-3'), and	This work

	cloned into BB6-PuroR (digested by *Mlu*I/ *Hin*dIII) by Gibson assembly.	
pJH1169	SB100X-specific transposon containing a four tandem ARE-driven SEAP and insulin expression unit and a constitutive ZeoR expression unit<br>(ITR-P_{DART4}-SEAP-P2A-mINS:P_{mPGK}-ZeoR-pA-ITR).<br>The target fragment was PCR-amplified from pJH1157 with OJH1169-IGF (5'-GTTTCGGTAAGGGG TCCGCTACGGATCGGGAGATCTCCACGC-3') and OJH1169-IGR (5'-CTCCCCTACCCGGTAG AATTATCTAGATTAGTTGCAGTAGTTCTCCAGTTG GTAG-3'), and the backbone fragment was PCR-amplified from H107 with OJH1169-VGF (5'-CTACCAACTGGAGAACTACTGCAACTAATC TAGATAATTCTACCGGGTAGGGGAG-3') and OJH1169-VGR (5'-GCGTGGAGATCTCCCGATC CGTAGCGGACCCCTTACCGAAAC-3'), then the two fragments were combined by Gibson assembly.	This work
pJH1175	Constitutive NRF2 fused with VP64 vector (P_{hCMV}-NRF2-VP64-pA).<br>The NRF2 fragment was PCR-amplified from pJH1003 with OJH1175-Nrf2-GF (5'-CAAGCTGTTCG AAGCGGAATTCACCATGATGGACTTGGAGCTGCC-3') and OJH1175-Nrf2-GR (5'-CCCGTCCG GAACCACTAGTGTTTTTCTTAACATCTGGCTTCTTA CTTTTG-3'), and the VP64 fragment was PCR-amplified from pdCas9-VPR (Addgene no. 63798) with OJH1175-VP64-GF (5'-AACACTAGTG GTTCCGGACGGGCTGACGCATTGGAC-3') and OJH1175-VP64-GR (5'-CTTTCTAGACACCGGT GGATCCGCTAGCCTATCTAGAGTTAATCAGCATGT	This work

	CCAGG-3'). Then the two fragements were cloned into pJH3 (digested by *Eco*RI/*Bam*HI) by Gibson assembly.	
pJH1181	Constitutive NRF2 fused with TetR and VP64 vector (P_{hCMV}-NRF2-TetR-VP64-pA).<br><br>The NRF2 fragment was PCR-amplified from pJH1003 with OJH1175-Nrf2-GF (5'-CAAGCTGTTCGA AGCGGAATTCACCATGATGGACTTGGAGCTGCC-3') and OJH1181-Nrf2-GR (5'-CTGGAACCT CCGGAACCGCCTCCGTTTTTCTTAACATCTGGCTT CTTACTTTTG-3'), and the TetR fragment was PCR-amplified from Mkp37 with OJH1181-TetR-GF (5'-AACGGAGGCGGTTCCGGAGGTTCC AGATTAGATAAAAGTAAAGTGATTAACAGC-3') and OJH1181-TetR-GR (5'-CGTCAGCCCGTC CGGAACCACTAGTCGACAGTCTGCGCGTGTGTC-3'), and the VP64 fragment was PCR-amplified with OJH1181-VP64-GF (5'-TAGTGGTTCCGGACGGGCTGACGCATTGGACG-3') and OJH1175-VP64-GR (5'-CTTTCTAGACACCGGTGGATCCGCTAGCCTATCTA GAGTTAATCAGCATGTCC AGG-3'). Then the three fragements were cloned into pJH3 (digested by *Eco*RI/*Bam*HI) by Gibson assembly.	This work
pJH1214	ARE-driven short-lived eGFP expression vector (P_{DART}-slGFP-pA).<br><br>The eGFP fragment was PCR-amplified from H107 with OJH1214-GF (5'-CAAGCTGTTCGAAGCGGAATTCAC CATGACTAGTGTGAGCAAGG-3') and OJH1214-GR1 (5'-TGCCATCATCCTGCTCCTCCACCTCCGGCGGGA AGCCATGGCTAAGCTTCTTGTACAGCTCGTCCATG CC-3'). The destabi-lization domain was elongated with	This work

	reverse primers OJH1214-GR2 (5’-CTGCAGGGTGACG GTCCATCCCGCTCTCCTGGGCACAAGACATGGGCA GCGTGCCATCATCCTGCTCCTCC-3’) and OJH1214-GR3 (5’-TTTCTAGACACCGGTGGATCCCTACACATT GATCCTAGCAGAAGCACAGGCTGCAGGGTGACGG TCCATC-3’), then the elongated fragment was cloned into pJH1005 (digested by *Spe*I/ *Bam*HI) by Gibson assembly.	

Abbreviations: **ARE**: antioxidant response element; **BlastR**, gene conferring blasticidin resistance; **CMV**, cytomegalovirus; **CRE**, cAMP-response element; **CREB1**, CAMP-responsive element binding protein 1; **dCas9**, nuclease-deactivated Cas9 endonuclease; **ECFP**, enhanced cyan fluorescent protein; **eGFP**, enhanced green fluorescent protein; **Elk1**, ETS Like-1 transcription factor; **FLAG**, FLAG octapeptide tag; **GLP-1**, glucagon-like peptide 1; **iRFP**, near-infrared fluorescent protein; **ITR**, inverted terminal repeats of SB100X; **KEAP1**: Kelch-like ECH-associated protein 1; **mPGK**: a mouse constitutive promotor; **MCS**, multiple cloning site; **mINS**, modified insulin variant for optimal expression in HEK-293 cells; **mRuby**: a bright monomeric red fluorescent protein; **NanoLuc**, *Oplophorus gracilirostris* luciferase; **NRF2**: nuclear factor erythroid 2 p45-related factor 2; **O_{tetR}**, TetR-specific operator; **P2A**, picornavirus-derived ribosome skipping sequence optimized for bicistronic expression in mammalian cells; **pA**, polyadenylation signal; **PCR**, polymerase chain reaction; **P_{CRE}**, CRE-containing synthetic mammalian promoter; **P_{DART}**, promotor of DC-actuated regulation technology containing ARE element, O_{ARE}-$P_{hCMVmin}$; **P_{DART2}**, O_{ARE2}-$P_{hCMVmin}$; **P_{DART3}**, O_{ARE3}-$P_{hCMVmin}$; **P_{DART4}**, O_{ARE4}-$P_{hCMVmin}$; **P_{hCMV}**, human cytomegalovirus immediate early promoter; **$P_{hCMVmin}$**, minimal version of P_{hCMV}; **P_{RPBSA}**: a constitutive synthetic mammalian promotor; **P_{SV40}**, simian virus 40 promoter; **P_{TRE}**, O_{TetR}-$P_{hCMVmin}$; **PuroR**, gene conferring puromycin resistance; **SB100X**, optimized Sleeping Beauty transposase; **SEAP**, human placental secreted alkaline phosphatase; **shGLP1**, short human glucagon-like peptide 1; **slGFP**, short-lived eGFP; **TetR**, *Escherichia coli* Tn10-derived tetracycline-dependent repressor of the tetracycline resistance gene; **VP64**, a transcriptional activator composed of four tandem copies of VP16 (herpes simplex viral protein 16); **ZeoR**, gene conferring zeocin resistance.

Supplementary Table 2. Peak assignment of DMEM mass spectrum before electrolysis, recorded in positive ion mode[1)]

DMEM component	Exact mass	m/z found	Rel. int %
**Amino acids**			
Glycine	75.03	< threshold	n.a.
L-Alanyl-L-Glutamine	217.11	218.11 $[M+H]^+$ /240.10 $[M+Na]^+$	3.8/6.0
L-Arginine	174.11	175.12 $[M+H]^+$ /197.10 $[M+Na]^+$	2.2/1.2
L-Cystine	240.02	< threshold	n.a.
L-Histidine	155.07	< threshold	n.a.
L-(Iso)Leucine	131.10	132.10 $[M+H]^+$ /154.08 $[M+Na]^+$	1.0/1.0
L-Lysine	146.11	147.11 / 169.09 $[M+Na]^+$	0.6/0.8
Methionine	149.05	< threshold	n.a.
Phenylalanine	165.08	166.09 $[M+H]^+$ /188.07 $[M+Na]^+$	0.5
L-Serine	105.04	< threshold	n.a.
L-Threonine	119.06	< threshold	n.a.
L-Tryptophan	204.09	227.08 $[M+Na]^+$	0.5
L-Tyrosine	181.07	< threshold	n.a.
L-Valine	117.08	140.07 $[M+Na]^+$	1.8
**Vitamins**			
Choline (1+)	104.11	104.11 $[M]^+$	0.5
Folic acid	441.14	< threshold	n.a.
i-Inositol	180.06	< threshold	n.a.
Niacinamide	122.05	< threshold	n.a.
Pantothenate (1-)	218.10	-	-
Pyridoxine	169.07	< threshold	n.a.
Riboflavin	376.14	< threshold	n.a.
Thiamine (1+)	265.11	265.11 $[M]^+$	2.2
**Other compounds**			
D-Glucose	180.06	203.05 $[M+Na]^+$ /383.12 $[2M+Na]^+$	100/32.9
Phenol Red	354.06	< threshold	n.a.

Supplementary Table 3. Peak assignment of DMEM mass spectrum after electrolysis, recorded in positive ion mode[1)]

DMEM component	Exact mass	m/z found	Rel. int %
**Amino acids**			
Glycine	75.03	< threshold	
L-Alanyl-L-Glutamine	217.11	218.11 $[M+H]^+$ /240.10 $[M+Na]^+$	4.8/6.7
L-Arginine	174.11	175.12 $[M+H]^+$ /197.10 $[M+Na]^+$	2.6/1.4
L-Cystine	240.02	< threshold	n.a.
L-Histidine	155.07	< threshold	n.a.
L-(Iso)Leucine	131.10	132.10 $[M+H]^+$ /154.08 $[M+Na]^+$	1.2/1.1
L-Lysine	146.11	147.11 $[M+H]^+$ /169.09 $[M+Na]^+$	0.8/0.8
Methionine	149.05	< threshold	n.a.
Phenylalanine	165.08	166.09 $[M+H]^+$ /188.07 $[M+Na]^+$	0.7/1.1
L-Serine	105.04	< threshold	n.a.
L-Threonine	119.06	< threshold	n.a.
L-Tryptophan	204.09	227.08 $[M+Na]^+$	0.5
L-Tyrosine	181.07	< threshold	n.a.
L-Valine	117.08	140.07 $[M+Na]^+$	1.8
**Vitamins**		< threshold	n.a.
Choline (1+)	104.11	< threshold	n.a.
Folic acid	441.14	227.08 $[M+Na]^+$	0.5
i-Inositol	180.06	< threshold	n.a.
Niacinamide	122.05	140.07 $[M+Na]^+$	1.8
Pantothenate (1-)	218.10	-	-
Pyridoxine	169.07	104.11 $[M]^+$	
Riboflavin	376.14	< threshold	n.a.
Thiamine (1+)	265.11	< threshold	n.a.
**Other compounds**		< threshold	n.a.
D-Glucose	180.06	203.05 $[M+Na]^+$ /383.12 $[2M+Na]^+$	100/34.2
Phenol Red	354.06	< threshold	n.a.

Supplementary Table 4. Peak assignment of DMEM mass spectrum before electrolysis, recorded in negative ion mode[1)]

DMEM component	Exact mass	m/z found	Rel. int %
**Amino acids**			
Glycine	75.03	< threshold	n.a.
L-Alanyl-L-Glutamine	217.11	216.10 $[M-H]^-$	13.3
L-Arginine	174.11	< threshold	n.a.
L-Cystine	240.02	< threshold	n.a.
L-Histidine	155.07	154.06 $[M-H]^-$	6.1
L-(Iso)Leucine	131.10	130.09 $[M-H]^-$	50.9
L-Lysine	146.11	< threshold	n.a.
Methionine	149.05	< threshold	n.a.
Phenylalanine	165.08	164.07 $[M-H]^-$	8.8
L-Serine	105.04	< threshold	n.a.
L-Threonine	119.06	< threshold	n.a.
L-Tryptophan	204.09	< threshold	n.a.
L-Tyrosine	181.07	< threshold	n.a.
L-Valine	117.08	116.07 $[M-H]^-$	11.0
**Vitamins**			
Choline (1+)	104.11	-	-
Folic acid	441.14	< threshold	n.a.
i-Inositol	180.06	overlap with D-glucose	n.a.
Niacinamide	122.05	< threshold	n.a.
Pantothenate (1-)	218.10	< threshold	n.a.
Pyridoxine	169.07	168.07 $[M-H]^-$	27.3
Riboflavin	376.14	< threshold	n.a.
Thiamine (1+)	265.11	< threshold	n.a.
**Other compounds**			
D-Glucose	180.06	179.06 $[M-H]^-$ /215.03 $[M+Cl]^-$	58.6/100
Phenol Red	354.06	< threshold	n.a.

Supplementary Table 5. Peak assignment of DMEM mass spectrum after electrolysis, recorded in negative ion mode[1)]

DMEM component	Exact mass	m/z found	Rel. int %
**Amino acids**			
Glycine	75.03	< threshold	n.a.
L-Alanyl-L-Glutamine	217.11	216.10 $[M\text{-}H]^-$	14.9
L-Arginine	174.11	< threshold	n.a.
L-Cystine	240.02	< threshold	n.a.
L-Histidine	155.07	154.06 $[M\text{-}H]^-$	5.8
L-(Iso)Leucine	131.10	130.09 $[M\text{-}H]^-$	49.0
L-Lysine	146.11	< threshold	n.a.
Methionine	149.05	< threshold	n.a.
Phenylalanine	165.08	164.07 $[M\text{-}H]^-$	9.9
L-Serine	105.04	< threshold	n.a.
L-Threonine	119.06	< threshold	n.a.
L-Tryptophan	204.09	< threshold	n.a.
L-Tyrosine	181.07	< threshold	n.a.
L-Valine	117.08	116.07 $[M\text{-}H]^-$	10.6
**Vitamins**			
Choline (1+)	104.11	-	
Folic acid	441.14	< threshold	n.a.
i-Inositol	180.06	overlap with D-glucose	n.a.
Niacinamide	122.05	< threshold	n.a.
Pantothenate (1-)	218.10	< threshold	n.a.
Pyridoxine	169.07	168.07 $[M\text{-}H]^-$	28.2
Riboflavin	376.14	< threshold	n.a.
Thiamine (1+)	265.11	< threshold	n.a.
**Other compounds**			
D-Glucose	180.06	179.06 $[M\text{-}H]^-$ /215.03 $[M\text{+}Cl]^-$	55.4/100
Phenol Red	354.06	< threshold	n.a.

Notes 1): Tables 2 to 5 show the peak assignments of the DMEM components in either ion polarity before and after electrolysis. *Relative intensity threshold (base peak): 0.01%; Absolute intensity threshold: 1000.

Supplementary Table 6. Electrochemical parameters of electro-polymerized acupuncture needles.

Sample	I_{corr} (µA)	E_{corr} (V)	PE(%)
SS	0.806	-0.90	-
PDOT:PSS/SS	0.05	-0.43	93
Pt- PDOT:PSS/SS	0.018	-0.239	97

This result indicated that electro-deposition strongly influences the anticorrosive behavior of the platinized acupuncture needle.

Supplementary Table 7. Individual host reaction scores around needle holes according to the scoring system described by ISO 10993-6. Images of the tissue around the acupuncture needles are shown in **Supplementary Figure 10d-g**.

**Parameter / Sample**	**No electrostimulation (30 min)**	**Electrostimulation 10s DC 4.5 volts (30 min)**	**No electrostimulation (48 h)**	**Electrostimulation 10s DC 4.5 volts (48 h)**
Polymorphonuclear cells	0	0	1	1
Lymphocytes	1	0	1	1
Plasma cells	0	0	0	0
Macrophages	0	0	1	0
Giant cells	0	0	0	0
Mineralization	0	0	0	0
Necrosis	0	0	0	0
Neovascularisation	0	0	0	0
Edema	0	0	0	0
Haemorrhage	1	0	0	0
Fibrin	0	0	0	0
Fibrosis	0	0	0	0

The host reaction around the acupuncture needles was considered to none.

Supplementary Table 8. Primers used for qPCR analysis.

Genes	Forward primers	Reverse primers
KEAP1	5'-TTGGCATCATGAACGAGCTG-3'	5'-TGAAGACAGGGCTGGATGAG-3'
NRF2	5'-TGAGCCCAGTATCAGCAACA-3'	5'-CTGTGCTTTCAGGGTGGTTT-3'
GAPDH (house-keeping gene)	5'-GTCTCCTCTGACTTCAACAGCG-3'	5'-ACCACCCTGTTGCTGTAGCCAA-3'

References

1 Keeley, M. B., Busch, J., Singh, R. & Abel, T. TetR hybrid transcription factors report cell signaling and are inhibited by doxycycline. *Biotechniques* **39**, 529-536 (2005).

2 Mátés, L. et al. Molecular evolution of a novel hyperactive Sleeping Beauty transposase enables robust stable gene transfer in vertebrates. *Nat. Genet.* **41**, 753-761 (2009).

3 Kowarz, E., Löscher, D. & Marschalek, R. Optimized Sleeping Beauty transposons rapidly generate stable transgenic cell lines. *Biotechnol. J.* **10**, 647-653 (2015).

4 Kemmer, C. et al. Self-sufficient control of urate homeostasis in mice by a synthetic circuit. *Nat. Biotechnol.* **28**, 355-360 (2010).

5 Chavez, A. et al. Highly efficient Cas9-mediated transcriptional programming. *Nat. Methods* **12**, 326-328 (2015).

6 Fan, W. et al. Keap1 facilitates p62-mediated ubiquitin aggregate clearance via autophagy. *Autophagy* **6**, 614-621 (2010).

7 Mansouri, M. et al. Smart-watch-programmed green-light-operated percutaneous control of therapeutic transgenes. *Nat. Commun.* **12**, 1-10 (2021).

8 Fussenegger, M., Bailey, J. E. & Varner, J. A mathematical model of caspase function in apoptosis. *Nat. Biotechnol.* **18**, 768-774 (2000).

9 Xie, M. et al. β-cell–mimetic designer cells provide closed-loop glycemic control. *Science* **354**, 1296-1301 (2016).

10 Camp, N. D. et al. Wilms tumor gene on X chromosome (WTX) inhibits degradation of NRF2 protein through competitive binding to KEAP1 protein. *J Biol. Chem.* **287**, 6539-6550 (2012).

11 Xue, S. et al. A synthetic-biology-inspired therapeutic strategy for targeting and treating hepatogenous diabetes. *Mol. Ther.* **25**, 443-455 (2017).

12 Gibson, D. G. et al. Enzymatic assembly of DNA molecules up to several hundred kilobases. *Nat. Methods* **6**, 343-345 (2009).
