# Supplementary material for: An electrogenetic interface to program mammalian gene expression by direct current
Source: Nat Metab. 2023 Jul 31;5(8):1395–407. doi: 10.1038/s42255-023-00850-7 (PMC10447240; doi:10.1038/s42255-023-00850-7)
Supplement: Supplementary file 2 — Reporting Summary [file 42255_2023_850_MOESM2_ESM.pdf]

nature portfolio

Corresponding author(s): Martin Fussenegger

Last updated by author(s): Jun 5, 2023

Reporting Summary

Nature Portfolio wishes to improve the reproducibility of the work that we publish. This form provides structure for consistency and transparency in reporting. For further information on Nature Portfolio policies, see our Editorial Policies and the Editorial Policy Checklist.

Statistics

For all statistical analyses, confirm that the following items are present in the figure legend, table legend, main text, or Methods section.

n/a	Confirmed	
☐	☒	The exact sample size (*n*) for each experimental group/condition, given as a discrete number and unit of measurement
☐	☒	A statement on whether measurements were taken from distinct samples or whether the same sample was measured repeatedly
☐	☒	The statistical test(s) used AND whether they are one- or two-sided<br>*Only common tests should be described solely by name; describe more complex techniques in the Methods section.*
☒	☐	A description of all covariates tested
☐	☒	A description of any assumptions or corrections, such as tests of normality and adjustment for multiple comparisons
☐	☒	A full description of the statistical parameters including central tendency (e.g. means) or other basic estimates (e.g. regression coefficient) AND variation (e.g. standard deviation) or associated estimates of uncertainty (e.g. confidence intervals)
☐	☒	For null hypothesis testing, the test statistic (e.g. *F*, *t*, *r*) with confidence intervals, effect sizes, degrees of freedom and *P* value noted<br>*Give P values as exact values whenever suitable.*
☒	☐	For Bayesian analysis, information on the choice of priors and Markov chain Monte Carlo settings
☒	☐	For hierarchical and complex designs, identification of the appropriate level for tests and full reporting of outcomes
☒	☐	Estimates of effect sizes (e.g. Cohen's *d*, Pearson's *r*), indicating how they were calculated

Our web collection on statistics for biologists contains articles on many of the points above.

Software and code

Policy information about availability of computer code

Data collection	RNA-seq data processing: trimmomatic (v 0.35) was used for alignment to the GRCh38 genome with hisat2 (v 2.1.0), samtools (v 1.9) was used to sort and index the alignment BAM files, and featureCounts from Subread package (v 2.0.1) was used to count reads in the gene ranges, using human Ensembl annotation v105.<br>Poly(3,4-ethylenedioxythiophene) polystyrene sulfonate (PEDOT:PSS) coating was done by anode deposition using an electrochemical workstation (CHI760E, v 20.4.0.0).<br>The DC electrodeposition was performed using a potentiostat (CHI760E, v 20.4.0.0).<br>The batteries were tested with a potentiostat (CHI760E, v 20.4.0.0).<br>The qPCR analysis was performed by QuantStudio 3 (QuantStudio Design & Analysis Software, ThermoFisher, v 1.5.1).<br>Supplementary movie 1 was filmed using a Huawei P30 mobile phone (ELE-L29, v 12.0.0). Supplementary movies 2 and 3 were recorded on a Zeiss LSM 980 Airyscan microscopic system (ZEN blue,v 3.5.093.00010).<br>The DC and AC voltages and currents were analyzed by connecting a potentiostat (CHI760E, serial no. E1174, v 20.4.0.0).<br>The electrochemical experiment was conducted with a potentiostat (CHI760E, serial no. E1174, v 20.4.0.0).

Data analysis	RNA-seq data analysis: The count vectors for all samples have been combined into a table, which was then subject to the secondary analysis in R. The quality control and sample consistency have been checked with PCA using R package PCATools (v2.6.0). The count table has been processed in the secondary (statistical) analysis with R scripts using edgeR (v3.32).<br>The western blotting images were analyzed using an enhanced chemiluminescence detection system (FusionPulse TS, cat. no. 121172301, v 5.12a).<br>The data were processed and determined by GraphPad Prism 8 (v 9.2.0, GraphPad Software Inc.) and Microsoft Excel (v 16.51, Microsoft Inc.).<br>The movies were further processed by Shotcut (v 21.09.20) and HandBrake (v 1.4.2) software.

For manuscripts utilizing custom algorithms or software that are central to the research but not yet described in published literature, software must be made available to editors and reviewers. We strongly encourage code deposition in a community repository (e.g. GitHub). See the Nature Portfolio guidelines for submitting code & software for further information.

Data

Policy information about availability of data

All manuscripts must include a data availability statement. This statement should provide the following information, where applicable:

- Accession codes, unique identifiers, or web links for publicly available datasets
- A description of any restrictions on data availability
- For clinical datasets or third party data, please ensure that the statement adheres to our policy

The authors declare that all the data supporting the findings of this study are available within the paper and its supplementary materials. All original plasmids listed in Supplementary Table 1 are available upon request. The sequences pJH1003 (GenBank accession no.: ON256650: https://www.ncbi.nlm.nih.gov/nuccore/ON256650), pJH1004 (GenBank accession no.: ON256651: https://www.ncbi.nlm.nih.gov/nuccore/ON256651), pJH1005 (GenBank accession no.: ON256652: https://www.ncbi.nlm.nih.gov/nuccore/ON256652), pJH1054 (GenBank accession no.: ON256653: https://www.ncbi.nlm.nih.gov/nuccore/ON256653), pJH1101 (GenBank accession no.: ON256654: https://www.ncbi.nlm.nih.gov/nuccore/ON256654) and pJH1169 (GenBank accession no.: ON256655: https://www.ncbi.nlm.nih.gov/nuccore/ON256655) are available on GenBank.

Research involving human participants, their data, or biological material

Policy information about studies with human participants or human data. See also policy information about sex, gender (identity/presentation), and sexual orientation and race, ethnicity and racism.

Reporting on sex and gender	No human participants involved in this study.
Reporting on race, ethnicity, or other socially relevant groupings	No human participants involved in this study.
Population characteristics	No human participants involved in this study.
Recruitment	No human participants involved in this study.
Ethics oversight	No human participants involved in this study.

Note that full information on the approval of the study protocol must also be provided in the manuscript.

Field-specific reporting

Please select the one below that is the best fit for your research. If you are not sure, read the appropriate sections before making your selection.

☒ Life sciences ☐ Behavioural & social sciences ☐ Ecological, evolutionary & environmental sciences

For a reference copy of the document with all sections, see nature.com/documents/nr-reporting-summary-flat.pdf

Life sciences study design

All studies must disclose on these points even when the disclosure is negative.

Sample size	In practice, the sample size used in this study is usually determined based on the need for it to offer sufficient statistical power, and the time, cost, or convenience of collecting the data. No statistical methods were used to predetermine sample size.
Data exclusions	No data were excluded from the analyses.
Replication	The data presentation, sample size of biological replicates (n), statistical analysis and significance of differences are shown in the figures. All in vitro experiments were reproduced at least twice, unless otherwise stated. For the mouse experiments, biological replicates (n = 5 mice) were used, unless otherwise stated. The details are described in each figure legend.
Randomization	All samples in this study were allocated randomly.

Blinding	All investigators involved in this study were blinded to group allocation during data collection and analysis.

Reporting for specific materials, systems and methods

We require information from authors about some types of materials, experimental systems and methods used in many studies. Here, indicate whether each material, system or method listed is relevant to your study. If you are not sure if a list item applies to your research, read the appropriate section before selecting a response.

Materials & experimental systems

n/a	Involved in the study
☐	☒ Antibodies
☐	☒ Eukaryotic cell lines
☒	☐ Palaeontology and archaeology
☐	☒ Animals and other organisms
☒	☐ Clinical data
☒	☐ Dual use research of concern
☒	☐ Plants

Methods

n/a	Involved in the study
☒	☐ ChIP-seq
☒	☐ Flow cytometry
☒	☐ MRI-based neuroimaging

Antibodies

Antibodies used	Anti-KEAP1, Abcam, Cat. no. ab227828, Western blot (1:5000), Lot. no. GR3397951-8;<br>Anti-NRF2, Abcam, cat. no. ab137550, Western blot (1:5000), Lot. no. GR3419093-1;<br>Anti-β-actin, Sigma cat. no. A2228, Western blot (1:5000), Lot. no.118M4829V;<br>Donkey anti-rabbit IgG (secondary), Sigma, cat. no. GENA934, Western blot (1:10000), Lot. no. 17528149;<br>Sheep anti-mouse IgG (secondary), Sigma, cat. no. GENA931V, Western blot (1:10000), Lot. no. 9739640.
Validation	All the commercially available antibodies used in this study were validated by the manufacturers and or previous publications through Western blot.<br>Anti-KEAP1 (https://www.abcam.com/keap1-antibody-epr22664-26-ab227828.html);<br>Anti-NRF2 (https://www.abcam.com/nrf2-antibody-ab137550.html);<br>Anti-β-actin (https://www.sigmaaldrich.com/CH/en/product/sigma/a2228);<br>Donkey anti-rabbit IgG (https://www.sigmaaldrich.com/CH/en/product/sigma/gena9341ml);<br>Sheep anti-mouse IgG (https://www.sigmaaldrich.com/CH/en/product/sigma/gena9311ml).

Eukaryotic cell lines

Policy information about cell lines and Sex and Gender in Research

Cell line source(s)	Cell lines used in this study: HEK-293, ATCC: CRL-11268; HT-1080, ATCC: CCL-121; HeLa, ATCC: CCL-2; hMSC-TERT (Simonsen et al., Nature Biotechnology, 2002), RRID:CVCL_Z015; BHK-21, ATCC: CCL-10; CHO-K1, ATCC: CCL-61; Hep G2, ATCC: CRL-11997; Caco-2, ATCC: HTB-37; C2C12, ATCC: CRL-1772.
Authentication	All the cell lines used in this study were re-authenticated by the supplier and the authorities of the Department of Biosystems Science and Engineering (D-BSSE) of the ETH Zurich in Basel, Switzerland. The quality of the cells was double-checked by the morphology before start of experiment.
Mycoplasma contamination	The authors confirmed that the cell lines in this study were tested for mycoplasma negative.
Commonly misidentified lines<br>(See ICLAC register)	No commonly misidentified cell lines were used in this study.

Animals and other research organisms

Policy information about studies involving animals; ARRIVE guidelines recommended for reporting animal research, and Sex and Gender in Research

Laboratory animals	The 6-8-week-old wild-type male Swiss mice (C57BL/6J, Janvier Labs) were used in this study. All mice were housed in a 12-hour light-dark cycle condition (5 mice per cage). The ambient temperature is 21±1°C with 50±10% humidity.
Wild animals	The authors declare that no wild animals were used in this study.
Reporting on sex	The male mice used in this study were randomly selected by following previous studies (Bai et al., Nature Medicine, 2019; Krawczyk et al., Science, 2020; Zhou et al., Nature Biotechnology, 2021; Chen et al., Nature Chemical Biology; Schneider et al., Science Advances, 2021), as well as due to the convenience of husbandry with the same sex. Sex was not considered in study design. No data disaggregated for sex were collected.

Field-collected samples	The authors declare that no field-collected samples were used in this study.
Ethics oversight	All experiments involving animals were performed in accordance with the Swiss animal welfare legislation, approved by the veterinary office of the Canton Basel-Stadt, Switzerland (license number: 2996/30779) and conducted by Shuai Xue (LTK4899) and Jinbo Huang (LTK5912) at the Department of Biosystems Science and Engineering (D-BSSE) of the ETH Zurich in Basel and according to the directives of the European Community Council (2010/63/EU), approved by the French Republic (project no. DR2018-40v5 and APAFIS no. 16753) and carried out by Shuai Xue, Jinbo Huang and Ghislaine Charpin-El Hamnri (no. 69266309) at the University of Lyon, Institut Universitaire de Technologie (IUT), F69622 Villeurbanne, France.

Note that full information on the approval of the study protocol must also be provided in the manuscript.
