# Supplementary figures and images for: An electrogenetic interface to program mammalian gene expression by direct current
Source: Nat Metab. 2023 Jul 31;5(8):1395–407. doi: 10.1038/s42255-023-00850-7 (PMC10447240; doi:10.1038/s42255-023-00850-7)

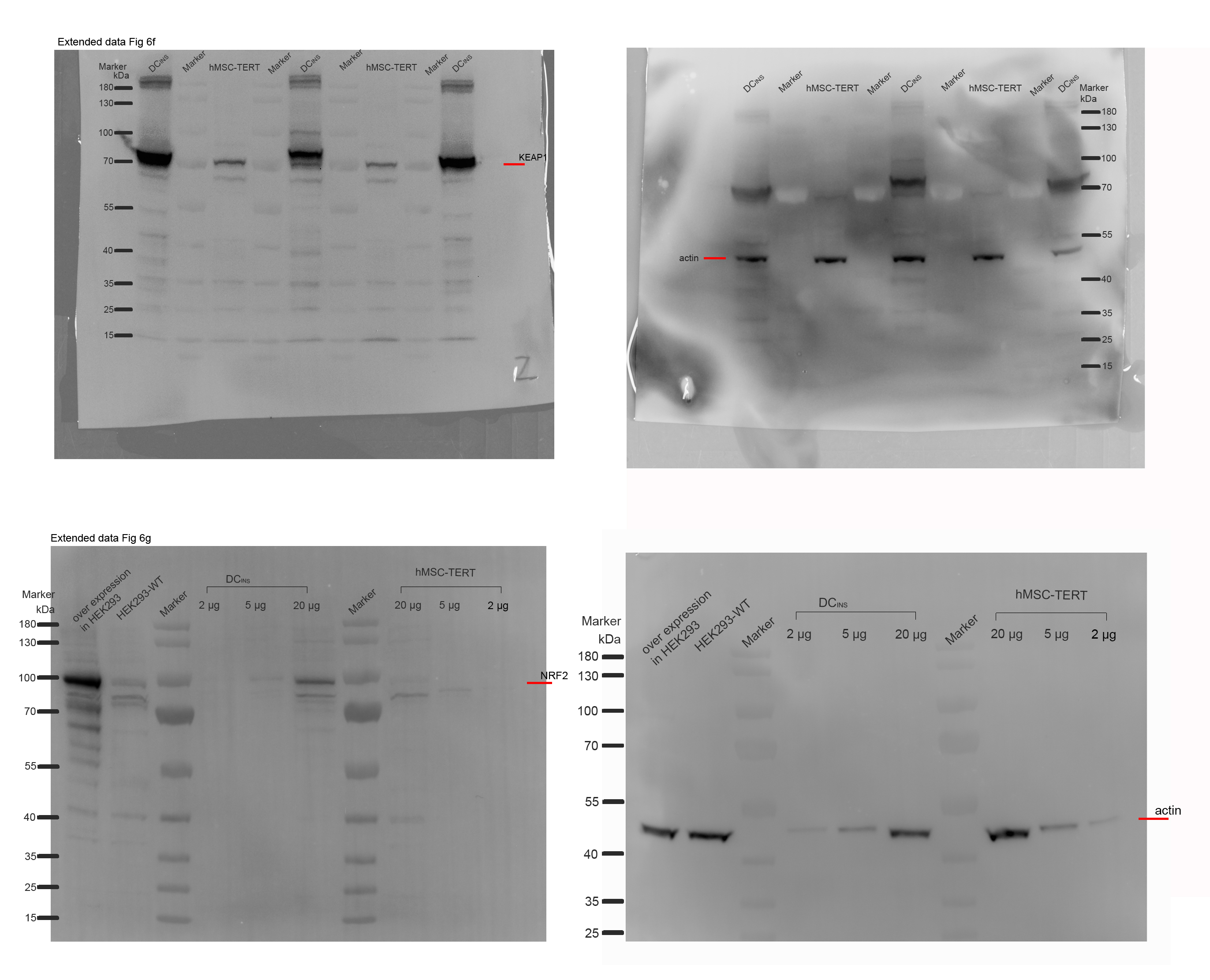

Supplement: Source Data Extended Data Fig. 6 — Unprocessed western blot gels. [file 42255_2023_850_MOESM18_ESM.jpg]
